# Psychoactive and other ceremonial plants from a 2,000-year-old Maya ritual deposit at Yaxnohcah, Mexico

**DOI:** 10.1371/journal.pone.0301497

**Published:** 2024-04-26

**Authors:** David L. Lentz, Trinity L. Hamilton, Stephanie A. Meyers, Nicholas P. Dunning, Kathryn Reese-Taylor, Armando Anaya Hernández, Debra S. Walker, Eric J. Tepe, Atasta Flores Esquivel, Alison A. Weiss

**Affiliations:** 1 Department of Biological Sciences, University of Cincinnati, Cincinnati, Ohio, United States of America; 2 Department of Plant and Microbial Biology, Biotechnology Institute, University of Minnesota, St. Paul, Minnesota, United States of America; 3 Department of Geography & GIS, University of Cincinnati, Cincinnati, Ohio, United States of America; 4 Department of Anthropology, University of Calgary, Calgary, Alberta, Canada; 5 Laboratorio de Geomática, CEDESU, Universidad Autónoma de Campeche, Mexico City, Mexico; 6 Florida Museum of Natural History (FLMNH), University of Florida, Gainesville, Florida, United States of America; 7 Programa de Posgrado en Estudios Mesoamericanos, Universidad Nacional Autónoma de México, Mexico City, Mexico; 8 Department of Molecular Genetics, Biochemistry and Microbiology, University of Cincinnati, Cincinnati, Ohio, United States of America; New York State Museum, UNITED STATES

## Abstract

For millennia, healing and psychoactive plants have been part of the medicinal and ceremonial fabric of elaborate rituals and everyday religious practices throughout Mesoamerica. Despite the essential nature of these ritual practices to the societal framework of past cultures, a clear understanding of the ceremonial life of the ancient Maya remains stubbornly elusive. Here we record the discovery of a special ritual deposit, likely wrapped in a bundle, located beneath the end field of a Late Preclassic ballcourt in the Helena complex of the Maya city of Yaxnohcah. This discovery was made possible by the application of environmental DNA technology. Plants identified through this analytical process included *Ipomoea corymbosa* (*xtabentun* in Mayan), *Capsicum* sp. (chili pepper or *ic* in Mayan), *Hampea trilobata* (*jool*), and *Oxandra lanceolata* (*chilcahuite*). All four plants have recognized medicinal properties. Two of the plants, jool and chilcahuite, are involved in artifact manufacture that have ceremonial connections while chili peppers and xtabentun have been associated with divination rituals. Xtabentun (known to the Aztecs as *ololiuhqui*) produces highly efficacious hallucinogenic compounds and is reported here from Maya archaeological contexts for the first time.

## Introduction

**Fig 1:**
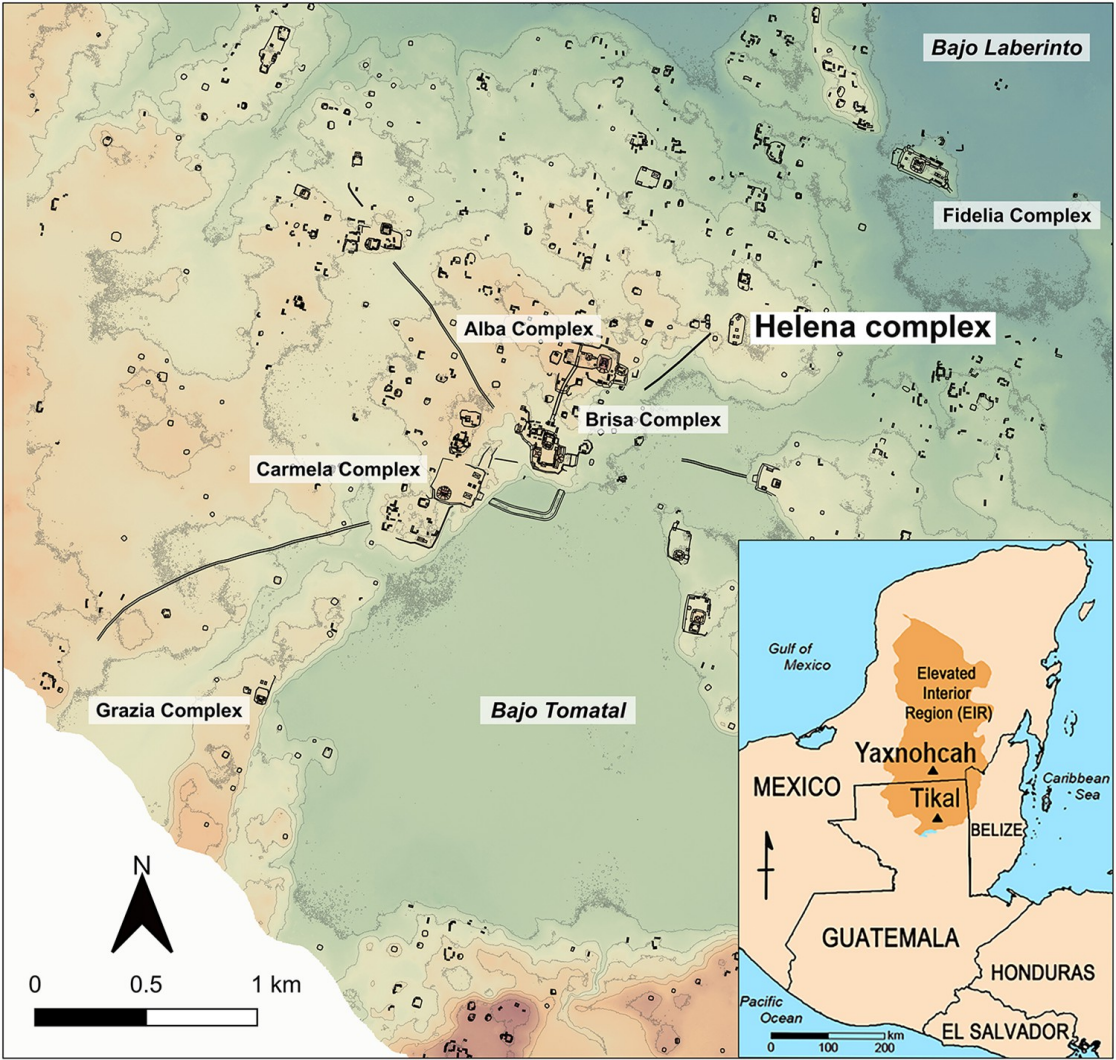
Map of Yaxnohcah showing the juxtaposition of the Helena complex to the Brisa complex and other ceremonial complexes within the site core. The inset in the lower right corner illustrates the location of Yaxnohcah within its regional context. Image created by David Lentz using Photoshop CS6, Vers. 13.0.1 × 64 (https://www.adobe.com/products/photoshop.html). Base map reprinted from [1] under a CC BY license, with permission from David L. Lentz, original copyright 2022).

In this paper we report on the analysis of a collection of plants, known in Mesoamerica for their ritual and medicinal properties, that were discovered beneath an early plaza floor of a civic ceremonial platform upon which was constructed a ballcourt at the ancient Maya city of Yaxnohcah. Remains of the ancient Precolumbian city were found in what is now the State of Campeche, Mexico, located 11 km north of the Guatemalan border and 15 km southeast of the ancient Maya city of Calakmul (Fig 1). Currently, the site is contained within the Calakmul Biosphere Reserve and is shrouded deep within the surrounding Neotropical forest.

Most of what is known about Maya rituals comes from modern ethnographic sources. Information about ancient maya ceremonial activities has been obtained from murals, scenes from ceramic vessels, stone carvings, four remaining codices (Maya books written on bark paper) and occasional well-preserved archaeological contexts. Blood-letting rituals are often discussed [2] but most ceremonial offerings are comprised of tobacco, incense, food or other plants with symbolic significance [3]. These rituals are performed to appease the Maya gods and maintain a balance in the cosmos or *k’ex*.

Archaeologically speaking, ritual contexts in the Maya area have been encountered, but it is a rare circumstance when the plants associated with a ritual activity are preserved and, if so, only a partial record can be detected. For the most part, the conditions for plant preservation in the wet-and-dry Neotropics are very poor [4]. A few exceptional contexts, however, have been discovered that are favorable for the preservation of ritual plants. These would include the Cerén site in central El Salvador, where a Late Classic volcanic eruption caused a Maya village to be covered with ash [5], cave sites where plant remains were preserved in ceramic vessels [6], water-logged sites such as the San Andres site in Mexico [7], in sealed contexts as found in offerings at Lamanai in Belize [8] and in other special circumstances. Otherwise, only partial recovery of identifiable plant materials is achievable.

Barring special circumstances, macrobotanical remains are most often encountered when they have been partially burned or carbonized [9]. Pollen evidence is often available due to the durability of the tough sporopollenin in the exine layer, but most plants in the Neotropics are zoophilous, pollinated by insects, birds, bats or other animals so the pollen from these species tends to be large and produced in small numbers. The wind pollinated, or anemophilous, pollen species produce abundant pollen, but represent only a fraction of the overall plant diversity in the Neotropics. Furthermore, pollen grains recovered often cannot be identified beyond the family taxonomic level, thus limiting their interpretive impact. Similarly, phytoliths, or silicacious bodies produced by some plants, are durable and can often be recovered when no other type of plant remains can be found. Because not all plants produce identifiable phytoliths and those that do often cannot be identified to species, this source of information, although in many cases very useful, has its limitations [10]. Starch grains [11] can be recovered from archaeological contexts too, such as whole vessels and from the surfaces of other artifacts, but only from special conditions and from a limited array of plants because many plants do not produce identifiable starch grains. Sometimes, the artifacts themselves will provide an indication of the substances involved in a ritual setting as with carved mushroom stones indicating the use of hallucinogenic mushrooms as a ritual component found at the Kaminaljuyu site in highland Guatemala [12]. Finally, plant chemical residues can be detected in some contexts if plants produce unusual compounds, such as theobromine in chocolate [13], although here again these tests can be administered only in unusual contexts with a finite array of outcomes possible.

In short, all means of recovery of plant remains from archaeological contexts are informative, but have shortcomings stemming from the various ways plant parts are produced, disseminated and preserved. Accordingly, the implementation of eDNA technology greatly augments the paleoethnobotanical toolkit and offers significant potential to fill in the gaps caused by the constraints encountered with other approaches. This new application can contribute substantially to our understanding of plants associated with Maya ritual features and this study at Yaxnohcah is an excellent example of how this technology can be applied.

## Materials and methods

### Study site

**Fig 2:**
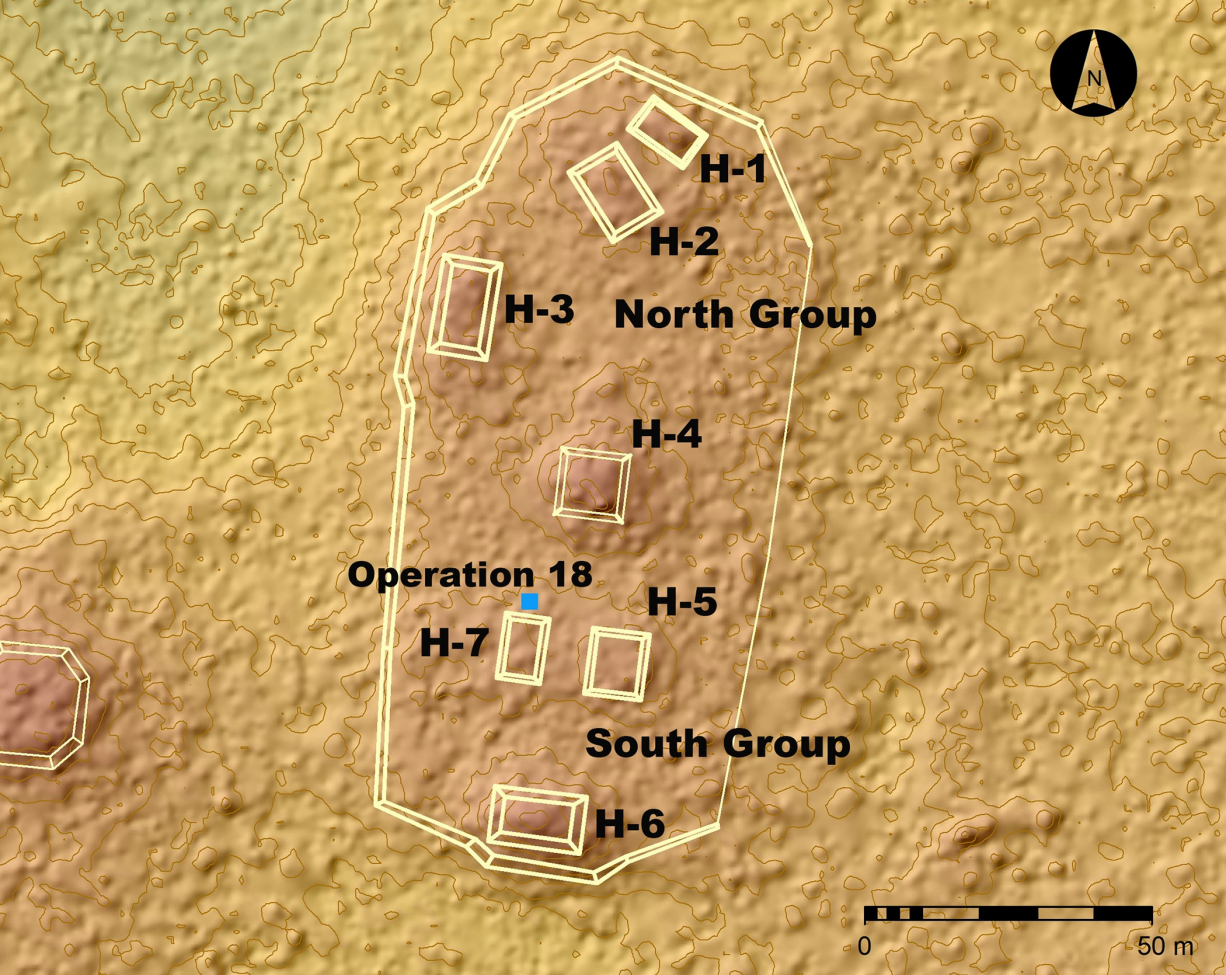
Map of the Helena complex showing the location of the excavation (Operation 18) in relationship to the structures of the ballcourt (Structures H-4 through H-7). H-7 is the westernmost lateral structure, or banquette, of the ballcourt. Image created by Atasta Flores Esquivel and David Lentz using Photoshop CS6, Vers. 13.0.1 × 64 (https://www.adobe.com/products/photoshop.html). Base map reprinted from [14] under a CC BY license, with permission from Verónica A. Vázquez López, original copyright 2022).

From 2016 to 2022, excavations were conducted at the Helena complex, a 1-meter high stone and earthen platform measuring 68 m east-west by 147 m north-south (Fig 2). Although modest in size, the Helena complex was linked by a causeway to the more elaborate Brisa E-group ceremonial complex located 900 m to the southwest, attesting to its importance within the Yaxnohcah community. The platform originally supported several Middle Preclassic (1000–400 BCE) domestic structures. Subsequently, during the Late Preclassic period (400 BCE-200 CE), the Helena platform was remodeled and a ballcourt was added [14].

**Fig 3:**
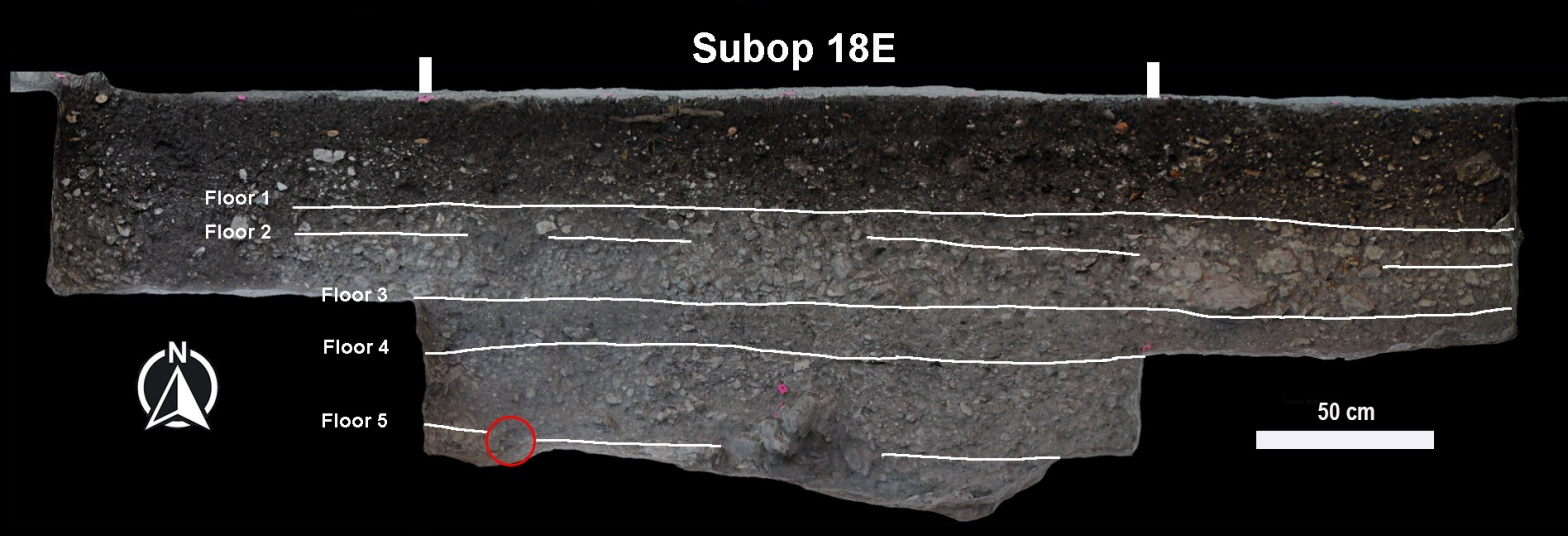
Photograph of the north profile of Operation 18. The location of the ceremonial deposit discussed in the text is encircled in red. Image created by Atasta Flores Esquivel and David Lentz. Reprinted from [17] under a CC BY license, with permission from Verónica A. Vázquez López, original copyright 2019).

Sediment was collected for environmental DNA (eDNA) analysis from a Preclassic stratum near the base of the platform where a dark, organic rich stain was observed (Fig 3). The archaeological eDNA technique has been applied successfully at the ancient Maya city of Tikal [15,16] and previously at Yaxnohcah [1]. The organic sample, labeled a “special deposit,” came from within the platform, 84 cm from the surface and articulated with the bottom of Floor 4 but intruded into Floor 5. While Floor 5 and the fill beneath it contained early Middle Preclassic ceramics (800–600 BCE) and represent the original bedrock leveling of the platform, the intrusive special deposit contained numerous Late Preclassic ceramics, lithics (including obsidian) and a figurine fragment.

The special deposit was associated with a substantial renovation of the Helena platform during the Late Preclassic period, when the character of the platform was transformed from domestic to ceremonial [14]. The location of the special deposit below the end field of the ballcourt and north of the western lateral structure clearly attests to its importance in the foundational rituals. A radiocarbon sample (Beta #613109) from the stratum associated with the special deposit produced a date of 158 cal. BCE—26 cal. CE (S1 Table), which is consistent with the Wob type ceramics recovered from the surrounding contexts (S2 Table).

### Paleoethnobotanical analysis

Paleoethnobotanical studies of ancient plant remains from Yaxnohcah have contributed to the reconstruction of environmental conditions of the ancient Maya as well as their ceremonial activities, dietary habits and other plant uses. Detailed descriptions of the materials and methods employed in this study, both in the laboratory and in the field, have been described in previous publications [1,15,16,18–21]. Samples designated for environmental DNA analysis were retrieved from all significant archaeological contexts at Yaxnohcah. The sediment for eDNA analysis described in this paper was extracted from a dark-stained area observed in Operation 18E9, a test pit located beneath the Helena ballcourt (Fig 2).

During fieldwork, sediments for eDNA analysis were collected in sterile, cryogenic, O-ring sealable containers half-filled with RNAlater (Invitrogen). The RNAlater inhibits enzymatic activity and acts as a preservative for the DNA contained within. All Yaxnohcah sediment samples were transported to lab facilities in the United States where they were stored in a– 80°C freezer. To avoid the possibility of contamination, DNA extractions were conducted in a Department of Biological Sciences laboratory that had been exclusively dedicated to the extraction of bacterial DNA, thus eliminating the chances of contamination by extraneous vascular plant DNA, the focus of our research.

Rigorous standard protocols designed to prevent the introduction of contaminating genetic material were utilized [22–24]. The clean bench and all equipment used under the hood (pipettes, tips, fresh receiving vessels, etc.) were exposed to UV light before and after extractions. Useable surfaces were cleaned with bleach and 70% ethanol after each use. Prior to DNA extraction, sediment samples were thawed to 4°C in a standard refrigerator before insertion into extraction tubes with glass beads, using sterile conditions, then sealed before insertion into a bead-beating machine (Biospec Products) for sample homogenization. Extraction protocols followed strict workflow guidelines employed in our previous studies and those of others [1,15,16,25–27]. The DNA sample examined in this study was extracted from archaeological sediments using a DNeasy Power Soil Kit manufactured by Qiagen [25,26].

Based on markers we selected, LGC RAPiD Genomics LLC (Gainesville, FL) designed genetic probes that were compatible with the capture-Seq protocol they use, in this case to capture plant DNA sequences from our extraction. Markers came from a wide phylogenetic breadth of plant taxa (S3 Table). We focused on nuclear and choloroplast gene sequences and employed genetic markers similar to those used in other ancient vegetation studies [28–30] to construct the genetic probes. Markers were selected that were known to be highly variable and/or well represented in Genbank, including both nuclear ribosomal ITS markers and plastid markers, e.g., *matK*, *ndhF*, *psbA-trnH*, *rbcL*, *rpl10*, *trnL-trnF*, *trnT-trnL*, and *ycf1*.

Design of the probe set was based on sequences from 104 species representing 54 genera across 33 families of vascular plants (S3 Table). The species we selected were known domesticated plants from the Western Hemisphere and characteristic wild plants from the Yucatan region. The Helena extract was subjected to equimolar pooling and was sequenced using an Illumina HiSeq 2X150 system. To reduce the risk of contamination, libraries were built with robot automation which essentially eliminated the possibility of human error during processing. Also, libraries were dual indexed. Dual indexing increases the accuracy of sample identification by adding an extra identifier to minimize issues caused by index-hopping, or index switching. Index hopping occurs when sequencing reads are assigned to incorrect indices, potentially leading to misalignment of reads or incorrect assumptions in downstream analyses. Using unique dual indices minimizes index hopping and is frequently employed for sensitive applications such as ours. Illumina sequence data were cleaned and trimmed with Trimmomatic (Vers. 0.36): a flexible trimmer for Illumina sequence data designed to remove adapters and improve quality by trimming with a sliding window of 10 at a quality score of 30. Cleaned reads were assembled into sequences using Meta-Spades.

Assembled sequences were screened against the target genes using the BLASTN algorithm associated with the U.S. National Center for Biotechnology Information (NCBI) database, or GenBank. Vascular plant sequences were separated from the large volume of bacterial and fungal sequences using this process. The vascular plant genetic sequence BLAST results we received from LCG Rapid Genomics were further processed by eliminating reads of plants that were not native to Central America using the Royal Botanic Gardens, Kew Plants of the World database (https://powo.science.kew.org).

### Ethics statement

Because the excavations took place on federal property in Mexico the project was conducted with permission from the Archaeology Council of the Instituto Nacional de Antropología e Historia (INAH permit #401.15.3-2017/1011 awarded to K.R.T. and A.A.H.) and the Comisión Nacional de Áreas Naturales Protegidas (CONANP permit # F00.9. D-RBC-046/2018 awarded to A.A.H. and K.R.T.). All sediment samples intended for eDNA analysis were brought into the US with permission from the US Department of Agriculture (USDA-APHIS Permit to Receive Soil #P330-19-00123 awarded to N.P.D.). In addition, Dr. Michael Linke, who serves as Chair of the University of Cincinnati Institutional Review Board, read our project description and, because there were no human subjects involved, determined that this project did not require UC IRB review and approval.

## Results

Overall, 105,577 gene sequences were captured and recorded. The vast majority of these sequences were from bacteria or fungi. Of these gene sequences, however, 15 were from vascular plants captured by our probe set. The size of vascular plant sequences ranged from 208 to 3692 bp, but most were short with a median sequence length of 263 bp. Eleven of the vascular plant DNA sequences were assigned to a broad taxonomic level, viz., family or order, likely because those genetic sequences were conserved across a wide range of taxa (see S4 and S5 Tables). Four of the eDNA plant sequences: i.e., *Ipomoea corymbosa* (L.) Roth, *Capsicum* sp., *Hampea trilobata* Standl. and *Oxandra lanceolata* (Sw.) Baill., however, could be identified to the genus or species level (Tables 1 and 2).

**Table 1:** Plants identified and the actual DNA sequences recovered.

Sequence/plant identified	Actual DNA sequence recovered
*Capsicum* sp.	TACACTTGAAAGATAGCCCATAAAGTTACGGGAATGGTTGGATAATTGGTTTATATGGAT CCTTCCTGTGTGAAAGCACAGAGAAAAATGACATTTCCAAAAATTGACAAGATAAAATTT CCATTTATTCATCAAAAGAAACGTCCCGTTTGAAGCCAGAATTGATTTTCCTTGATACCT AACATAATGCATGAAAGGATCCTTGAATAACCATAAGGTAACCTGAAAATCCTTAGCAAA GACTTCTACAAGACGTTCTATTT
*Ipomoea corymbosa*	TACAATTCATATTAGAGCGCAAAAAAATATTAAAATCTCAGAATTTACGATCAATTCATT CAATATTTCCTTTTTTAGAGGACAATTTCTCACATTTAAATTATGTATTAGATATACTAA TACCCTACCCCCCTCATCTGGAAATCTTGGTTCAAACTCTTCACTATTGGGTGAAAGACG CTTCTTCTTTGCATCTATTACGATTCTTTCT
*Hampea trilobata*	GGTAAAGGTTTTTCTCATGAGTAAATTATCGATTTTTTTATGAGTCCTAATTATTAGTCA TTCCCTTTATGGGTTCGACATGAATGTGTAGAAGAAGCAGTATATTGATAAATAAAAGAT ATTTTTTTCCAAAATCAAAAGAGCGATTGGGTTGAAAAAATAAAGGATTTCTAACCGTCT TCTTATCCTATAACGAACATAAATCAATTAGATGGCAAAAGATAGGATAGAGAATTCGTT GATGAATCTGCCTGTCTCCGAGGTATCTATTCTTTTCTTACTA
*Oxandra lanceolata*	TCTATCTGTATCAATATGCTTGTTCTCATCCAAAAAATGCACACAGTTCCTTATGTCGTA GCCGTCGACCAATACTGGATTTCTCTCCACAACTCTCCCAATTTCAGAATTAAAACAAAT TCGAATTCTCAATTCTCTACGACGTCTAGTAGATGGAATATTTTCAGGAACAAGCAAATC ATATTTTTTTTCTCGGCATCTTTGATTAGTTTGGTACTTATTCTTATGGACCAAGAAAAT ACCTA

**Table 2:** Plant sequence name and BLAST results.

Scientific Name	Sequence Length (bp)	Max Score	Total Score	Query cover	E-value	Percent ID	Accession Length	Accession Number
*Capsicum* sp.	263	479	479	99%	5e-131	99.63%	834	MN167200.1
*Ipomoea corymbosa*	211	390	390	100%	2e-104	100%	161102	KF242504.1
*Hampea trilobata*	283	518	518	100%	1e-142	99.65%	2499	KT966969.1
*Oxandra lanceolata*	245	420	420	100%	3e-113	97.55%	1556	GU937368.1

In general, ancient DNA tends to be degraded into fragments, typically of 40–500 bp [31]. The four sequences that are the focus of this paper are all between 200–300 bp, well within the expected range. Also, old DNA tends to have more of the pyrimidine thymine on the ends of the sequences [31]. All of the four sequences that are the focus of this study have thymine as the end pyrimidine on both ends of the molecules (Table 1). In addition, the conditions for preservation of eDNA are favorable in this context. First, the clay content is very high, ranging from 70–80 percent. Second, this feature was from an essentially sealed context because it was beneath, not one, but four paved floors. Therefore, the chance of percolation through those floors and layers of clay would be extremely low.

Each of four plants identified has special properties that would have made them a likely component of an ancient ritual. Together they form an intriguing set of medicinal and ceremonial plants whose combination elicits questions about symbolic meaning and religious associations. Parenthetically, two of those vascular plant sequences assigned to a broader taxonomic level were found in copal (*Protium copal* [Schltdl. & Cham.] Engl.) and maize (*Zea mays* L.), also likely components of a ceremonial offering, but are not discussed herein because we could not exclude closely related plants known to have those same gene sequences.

A similar feature to the ceremonial deposit at the Helena ballcourt, both in terms of context and time of deposition, was found inside a chultun near the Fidelia complex at Yaxnohcah. Chultuns are underground features that are frequently associated with Maya ceremonial activities and this particular feature was no exception. The deposit was super-positioned over a burial with a large number of artifacts including ceramics, obsidian objects, half of a bivalve seashell, and figurines. The largest number of DNA sequences (8) found in the sample came from the genus *Ehretia*. Also, there was a sequence from the genus *Ficus*. The bark of both of these genera are believed to have been sources of fibers for making paper by the ancient Maya [32]. The cluster of artifacts and human remains provide a strong indication that this was a ceremonial cache and the *Ehretia* and *Ficus* sequences may well have represented the remains of bark cloth, a blood-letting ritual, a paper headdress, or possibly a codex (an ancient book of the Maya). Any of these items would have been appropriate grave offerings for a scribe, priest or even a ruler [33,34]. Other similarities shared by the chultun cache and the Helena ballcourt feature is that they both were placed beneath a floor in a ceremonial context during the Late Classic period.

Genetic data generated by this project are available from the NCBI under BioProject number PRJNA804249, Biosample #SAMN25719082. Site reports and other basic data from the Yaxnohcah project are available online through the following link: https://www.mesoweb.com/resources/informes/Yaxnohcah2016.html.

## Discussion

**Fig 4:**
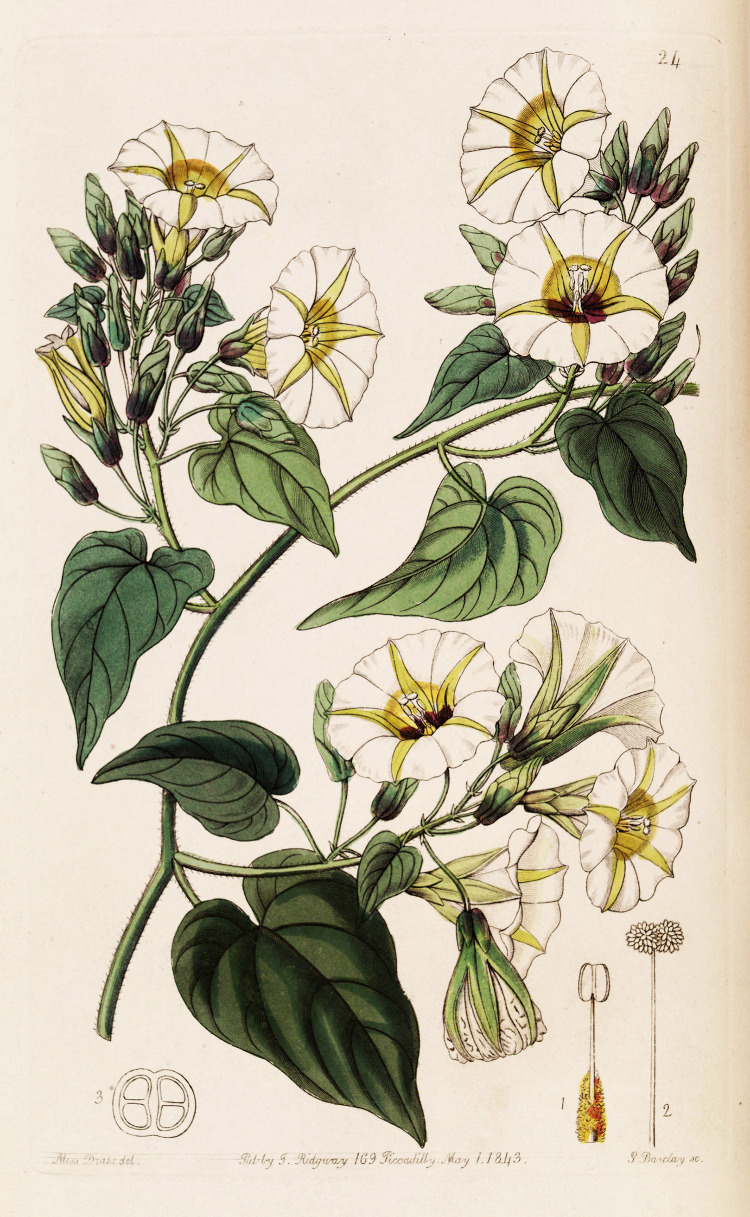
Print of *Ipomoea corymbosa* from Edwards Botanical Register. Reprinted from [43] under a CC BY license with permission from the Peter H. Raven Library, Missouri Botanical Garden, original copyright 1843.

Of the four plants species recovered from the dark-stained organic feature, the *I*. *corymbosa* (called *xtabentun* by the Yucatec Maya), a vine in the Convolvulaceae or morning glory family, is perhaps the most intriguing (Fig 4). Today, it is a common Maya practice to brew mead from the honey of bees fed on pollen from xtabentun flowers [35]. This alcoholic beverage is still commonly served in the cafes of southern Mexico. Xtabentun is known to the modern Maya as a ritual plant, but the description of its involvement in ritual practices is generally vague [36]. Colonial persecution arising from the use of xtabentun [37] may explain the lack of specific information about the way it has been used or discussed in post-European contact times. By contrast, the use of the plant among the Aztecs, who called it *coaxihuitl* or snake plant [38], has been well-documented by Hernandez [39] and other Spanish chroniclers [40]. The snake plant seeds, or *ololiuhqui* in Nahuatl (the language of the Aztecs), contain alkaloids with indoles of d-lysergic acid amide and d-lysergic acid methylcarbinolamide. These compounds are similar in structure and activity to ergoline derivatives of lysergic acid diethylamide (LSD) and alkaloids found in ergot fungi (*Claviceps* spp.) [41]. The seeds, with their hallucinogenic properties, were used in ceremonies by Aztec priests to create hallucinogenic visions that facilitated the communion with their gods [42]. These ceremonial interactions were deemed satanic by Spanish clerics and practitioners of divination ceremonies involving the native plant were widely persecuted, a circumstance which drove the ololiuhqui cults into hiding [41].

Because xtabentun was utilized by other Native American groups in Mesoamerica for ritual intoxication and divination, many believe that the Maya employed the plant for similar purposes [12,44–46]. Although details about the use of xtabentun among the ancient Maya are lacking, there are indications in the ethnographic literature that the Maya may have used the plant in much the same way the Aztecs did. For example, the Dresden codex, one if the four remaining books of the ancient Maya (written around 1200 CE on *Ficus* paper), has images of the xtabentun cordate leaves in Almanacs 13 and 23 associated with the diving death god (God A) and Chaac, respectively [44]. The close association between these prominent gods and the hallucinogenic plant likely had ritualistic significance. In the books of *Chilam Balam* (“Jaguar Priest” in Mayan), a set of volumes written by Maya scribes during the early Colonial period, there are passages that describe a *chilan*, or prophet, in a hallucinogen-induced trance lying face down with his mouth to the ground and speaking unintelligibly. Thompson [45] opines that the chilan story may have involved xtabentun as the hallucinogen. Xtabentun also has been listed as an ingredient in *balche*, the sacred alcoholic beverage of the Maya [34]. In short, it has long been hypothesized that the ancient Maya employed xtabentun [45,46] but prior to this study, there have been no reports of the plant in the Maya archaeological record [47,48].

Chili peppers (*Capsicum* sp. or *ic* in Yucatec and Tzeltal Mayan) in the Solanaceae or nightshade family, are thought of as a condiment or flavoring by most Old World societies, but the plant has a much greater depth of meaning for the Maya [49–52]. The earliest domesticated chili seeds (*Capsicum annuum* L.) were identified from El Riego phase deposits in the Tehuacan Valley, Mexico and dated to around 7,000 BP [53] indicating that the cultivation of chili peppers has an ancient history in Mesoamerica. Archaeobotanical evidence reveals that the use of chilis was not only of great antiquity but also was widespread among the ancient Maya [47]. The earliest chili pepper evidence from the Maya Lowlands was found in Early to Middle Preclassic contexts (1100–900 BCE) at the Cuello site in what is now northern Belize [54]. The best-preserved chili peppers from the Maya area, however, came from Late Classic deposits (ca. 600 CE) at the Cerén site in El Salvador where dried and carbonized fruits were found in kitchens, storehouses and ceramic vessels [55]. Although there are three closely related species of domesticated chili peppers from Central America (*C*. *annuum*, *C*. *frutescens* and *C*. *chinense*), only *C*. *annuum* was found in Mexico at the time of European contact [56]. Furthermore, it is clear that *C*. *annuum* was domesticated in Mexico [57]. Because of these reasons, in all likelihood the gene sequence recovered from the Helena context at Yaxnohcah was from *C*. *annuum*, even though the sequence is found in all three species.

Today, chili pepper seeds and fruits are used by the Maya as a treatment for an impressive array of diseases including tuberculosis, delayed parturition, diarrhea, blood in the stool or urine, earache, hemorrhoids, skin sores, arthritis, and asthma [58–61]. Faust and Lopez [62] record that Yucatec Maya healers bury chili pepper fruits in a hole dug into the floor of a home to ward off illness and to expel “bad winds” that may be the cause of disease. In addition to the dietary and medicinal uses, the Maya have employed chilis in a broad range of religious ceremonies such as the ritual of the *Bacabs* (Maya deities) by the Yucatec Maya [61] and the god pot renewal, first fruit and *u horɂor ik* ceremonies by the Lacandon Maya [63]. Chili peppers are often incorporated into Yucatec food bundles for ceremonies and festivals [64]. These pungent fruits also are added to the mix when the Lacandon brew their balche, an essential beverage in almost all of their rituals [63]. Similar to the Faust and Lopez observations [62], Maya shamans and sorcerers use chilis to prevent future illnesses and negative outcomes caused by a disharmony with one’s surroundings or for protection against malevolent witchcraft [58,65]. In addition, chili peppers are listed in the Dresden Codex as part of a “shamanic divination” ritual [43].

*Hampea trilobata*, or *jool* in Yucatec Mayan, is a small tree in the Malvaceae, or mallow family, common in the forests of the lowland Maya. It is the only species of the genus *Hampea* that is native to the Yucatan peninsula [66]. The bark and leaves of the tree are used to wrap food bundles for Maya ceremonies [64]. Furthermore, the Itza Maya fashion twine from beaten strips of the bark to manufacture baskets that serve as food containers [67]. In addition to its utility in making twine and cordage, the inner bark of jool is used to treat snakebites and scorpion stings [68].

*Oxandra lanceolata*, known as *chilcahuite* in southern Mexico, is a tree in the Annonaceae, or soursop family [69]. In English, the tree is called lancewood and in Spanish it is referred to as *palo de lanza* [70]. As the names suggest, it is used for making spears and also archery bows and fishing rods [71]. In addition, the plant has notable medicinal properties. One of the compounds found in oil extracted from the leaf, spathulenol, is a potent vasodilator and anesthetic. Another compound in the leaves of *O*. *lanceolata*, α-pinene, is a highly effective anti-biotic agent [72]. Thus, chilcahuite has many qualities that would have been useful to the ancient occupants of Yaxnohcah.

Although it is a considerable challenge to interpret a collection of plants from archaeological contexts through the opaque lens of 2,000 years of prehistory, the ethnographic, historical and archaeobotanical records offer substantial insight into the importance of this particular set of plants retrieved from the same context. The first salient point relevant to this discussion about the ancient Maya is that they were deeply religious and their interactions with the deities permeated almost every aspect of their existence [3]. They believed that the world was created by their gods and its preservation required relentless efforts by all humans to take an active part in rituals to maintain social order and, in a larger sense, the cosmic balance. Failure to maintain this balance would lead to disease, drought, or social strife [73]. Furthermore, basic human survival was thought to depend completely on the maintenance of harmonious relationships with the deities. Failure to do so would bring about relentless punishment [74]. The gods must be satisfied with appropriate rituals performed regularly. The Yucatec Maya, for example, practice a “fix earth” ceremony whereby guardian spirits are invoked to remove malignant spirits from a structure or domestic space [74]. This ceremony renders the space safe to inhabit.

Because of its hallucinogenic properties, the xtabentun find suggests that the purpose of this unusual collection may have been connected with divination. We do know that, in general, the consumption of hallucinogenic substances played a vital role in the rituals carried out by Maya kings and high priests because it empowered them to receive the energy of the gods [3]. These acts of divination were essential to the entire ritual system because they enabled the supplicant to understand the wishes of the gods so they might ascertain what kind of subsequent ceremonies should be planned to appease the deities most effectively [63]. The use of xtabentun as a component in making balche and as a medicinal plant are also suggestive that these may have been reasons why the plant was included in the special deposit.

The presence of chili peppers indicates that the deposit may have been part of a healing ceremony, a food offering, a component in balche, or as part of a divination ceremony. According to Maya cosmological tradition, houses and other structures are animate entities that require adequate sustenance. The act of “feeding” a structure is not merely ceremonial or an act of artistic expression, but a requirement at the time of construction [75]. The inhabitants must provide nourishment for the newly formed structure, otherwise the occupants will suffer illness [76]. The provision of nourishment for the emergent entity and the accompanying dedication rituals have been referred to as “ensouling ceremonies” [75]. Curiously, these kinds of rituals became more prevalent in the Maya area during the Late Preclassic period. For example, evidence of ensouling rituals was observed at K’axob and Cuello plaza spaces built on large basal platforms [75,77]. In these situations, it was thought that adequate propitiation of the gods was necessary to insure the health and prosperity of the inhabitants. Chili peppers were known to have been involved in all these kinds of Maya rituals and their presence in the Helena complex special deposit is significant.

The jool component likely represented some kind of containment vessel where the leaves or bark fibers were used to wrap the components into a bundle. If the components we observe in the ceremonial deposit were wrapped in a bundle, it would explain why so many ceremonial ingredients were found in such a constricted area. Following this line of thought, the other three components may have been clustered in a bundle or placed in a basket made of jool fibers or leaves. Another interpretation may involve the inclusion of jool because of its medicinal properties.

The chilcahuite evidence represents a more complex component that ties in well with the location of the special deposit beneath a ballcourt. For the Maya, activities in the ball game, hunting and warfare were all variations of the same activity. For example, the verb “jatz” in Mayan is used for throwing a spear and for hitting a ball [3]. The Lacandon and Tzotzil Maya incorporate miniature bows into the *mekchur* and other ceremonies [63,78]. Although chilcahuite is used for making archery bows, it is highly unlikely that this is what was represented in the Yaxnohcah special deposit because the use of bows and arrows had not been introduced into the Maya area until the Postclassic period [79] over a thousand years after the Late Preclassic bundle at Yaxnohcah was deposited. The reference is included here because the use of spears in a Preclassic ceremony may have been analogous to activities involving bows by the modern Maya who no longer use spears for hunting or warfare. Alternatively, the leaves of chilcahuite may have been incorporated into the bundle because of their medicinal properties.

## Conclusions

If the indications from ethnographic accounts are applied to the collection of plants found in the Helena ceremonial deposit, then a pattern of application emerges that provides a reasonable interpretation of this unusual find. The first connection between these four plants is that they all have medicinal applications and may have been combined in some kind of curative ceremony. The location of the ceremonial plant cluster, inserted below the 4^th^ floor of the platform at the base of a major renovation, suggests an association with the transformation of the platform from a domestic to a ceremonial space. As we have seen, the Maya are known to place healing bundles below floors as a protective measure to ward off external causes of illness [62].

An even stronger possibility, however, was that this was part of an ensouling or fix earth ritual designed to propitiate the gods in a way that would ensure their blessings in subsequent activities associated with the newly constructed ceremonial ballcourt space [80]. Finally, this assemblage of plants may have been part of a divination ritual. The chili peppers and the xtabentun, both used by Mesoamerican cultures in divination ceremonies, argue for this interpretation. Similarly, a recent discovery of an ancient ritual bundle in South America [81] bearing evidence of *Ipomoea corymbosa* represents a distant and less ancient, but strikingly parallel context. Whatever the intent of the Maya petitioners, it seems clear that some kind of divination or healing ritual took place at the base of the Helena ballcourt complex during the Late Preclassic period. On a final note, as with the ceremonial plants found at Yaxnohcah, a greater understanding of the ritual and other sacred practices of ancient cultures can now come into clearer focus with the assistance of eDNA evidence, a methodology whose promise for archaeology is only beginning to be explored.

## Supporting information

S1 Table AMS Radiocarbon dates from Operation 18. Dates in blue are most relevant to the ritual deposit.
(DOCX)

S2 Table Yaxnohcah chronology based on ceramic complexes. (DOCX)

S3 Table Species, markers, and sequences (available via the Genbank accession numbers) used to create the custom probeset for LGC Rapid Genomics’ Capture-seq pipeline. Unavailable sequence data is indicated by “—“.
(DOCX)

S4 Table Vascular plant nucleotide sequences from Operation 18E9 of the Helena complex. The following sequences were subjected to a BLAST search using the National Center for Biotechnology Information (NCBI) database. Unlike the four plants discussed in the main body of the paper, the plants listed in this table could not be resolved to the genus or species level, likely because the sequences we recovered were too highly conserved among plant taxa. The actual sequences for each plant listed here are followed by the results of the BLAST searches (S5 Table).
(DOCX)

S5 Table BLAST results from eDNA data. A decision tree for interpreting BLAST results for our eDNA data was published previously [16]. In brief, our eDNA gene sequences were compared to the NCBI database and their BLAST program (using a BLASTN algorithm) evaluated the closest fit for our gene sequences by generating Max scores and E values (Expect value). Generally, the higher the Max score the closer the match of our unknown sequences to sequences from plant species stored in the database. The E value can be stated as the likelihood that a specific sequence alignment is a result of chance rather than a true biological relationship between the unknown sequence and the species represented in the reference sequence. Thus, a lower E value indicates a more significant alignment. In numerous cases, only one plant species would be listed with the highest Max score. If the plant identified was native to southeastern Mexico or was a known New World cultigen, then our identification was clear and definitive. Because gene sequences can be conserved among even distantly related plants, however, it was not uncommon to find more than one species with a top ranked Max score. When this occurred, we followed the published decision tree to decide if a species or genus identification could be determined. Note that in all cases, we took a conservative approach and assigned a broader taxon if there was any ambiguity.
(DOCX)
