# Supplementary material for: Psychoactive and other ceremonial plants from a 2,000-year-old Maya ritual deposit at Yaxnohcah, Mexico
Source: PLoS One. 2024 Apr 26;19(4):e0301497. doi: 10.1371/journal.pone.0301497 (PMC11051596; doi:10.1371/journal.pone.0301497)
Supplement: S1 Table — Dates in blue are most relevant to the ritual deposit. (DOCX) [file pone.0301497.s001.docx]

**S1 Table.** **AMS Radiocarbon dates from Operation 18.** Dates in blue are most relevant to the ritual deposit.

| **Radiocarbon Sample #** | **Provenance** | **Context** | **Material Tested** | **Date Excavated** | |  | **Age**  **BP** | | | **2 Sigma**  **Range** | | | | **95% Calibrated**  **Dates** | | **Ceramic**  **Assoc.** | | | | |
| --- | --- | --- | --- | --- | --- | --- | --- | --- | --- | --- | --- | --- | --- | --- | --- | --- | --- | --- | --- | --- |
| 19C/0914 | OP18E-06 | Floor 3 |  | 2017 | |  | 1720 | | | | 30 | | 240–400 cal AD | | | | Chay | |  |  |
| Beta-613109 | OP18E-09 | carbonized material  in carbonate | charred material | 2017 | |  | 2050 | | | | 30 | | 158 cal BC–26 cal AD (95%)  50–55 cal AD (0.6%) | | | | Wob | |  |  |
| Beta-613108 | OP18D-19 | Sample # 6 | charred material | 2017 | |  | 2090 | | | | 30 | | 196–185 cal BC (1.3%)  178–38 cal BC (91.2%) 13 cal BC–4 cal AD (2.8%) | | | | late Macal  modes | |  |  |
| 19C/0918 | OP18D-24 | fill above bedrock | charcoal | 2017 | |  | 2170 | | | | 30 | | 360–150 cal BC (92.9%) 140 cal BC–110 cal AD (2.5%) | | | | Macal  mode | |  |  |
| Beta-613111 | OP18D-07 | lot matrix D7 | charred material | 2017 | |  | 2180 | | | | 30 | | 364–150 cal BC (94.3%) 131–121 cal BC (1.1%) | | | | Chay | |  |  |
| 17C/1223 | OP18C-12 | fill context | charcoal | 2016 | |  | 2310 | | | | 30 | | 410–360 cal BC (85.8%) 290–240 cal BC (9.6%) | | | | Macal | |  |  |
| 19C/0917 | OP18E-15 | lens above bedrock | charcoal | 2017 | |  | 2540 | | | | 30 | | 800–730 cal BC (44.4%) 690–660 cal BC (12.2%) 650–540 cal BC (38.9%) | | | | late  Macal | |  |  |
|  |  |  |  | |  | | |  |  | | |  | | |  | | |  | |  |
