# Supplementary material for: Psychoactive and other ceremonial plants from a 2,000-year-old Maya ritual deposit at Yaxnohcah, Mexico
Source: PLoS One. 2024 Apr 26;19(4):e0301497. doi: 10.1371/journal.pone.0301497 (PMC11051596; doi:10.1371/journal.pone.0301497)
Supplement: S2 Table — (DOCX) [file pone.0301497.s002.docx]

**S2 Table.** **Yaxnohcah chronology based on ceramic complexes.**

| Date estimates | Period name | | Period abbreviation | | Yaxnohcah ceramic complex | |
| --- | --- | --- | --- | --- | --- | --- |
| 1400-1500 CE | | Late Postclassic | | LPC | Luch |  |
| 1000-1400 CE | | Postclassic | | PC | Luch |  |
| 850 – 1000 CE | | Terminal Classic | | TC | Xichinche’ |  |
| 500 – 850 CE | | Late Classic | | LCP | Tux |  |
| 200 – 500 CE | | Early Classic | | EC | Kiwi’ |  |
| 100 BCE – 200 CE | | Terminal Preclassic | | TPreC | Wob |  |
| 300 – 100 BCE | | Late Preclassic | | LPreC | Chay |  |
| 600 – 300 BCE | | Late Middle Preclassic | | LMPreC | Um |  |
| 900 – 3000 BCE | | Early Middle Preclassic | | EMPreC | Macal |  |
