# Supplementary material for: Psychoactive and other ceremonial plants from a 2,000-year-old Maya ritual deposit at Yaxnohcah, Mexico
Source: PLoS One. 2024 Apr 26;19(4):e0301497. doi: 10.1371/journal.pone.0301497 (PMC11051596; doi:10.1371/journal.pone.0301497)
Supplement: S3 Table — Unavailable sequence data is indicated by “—“. (DOCX) [file pone.0301497.s003.docx]

**S3 Table.** **Species, markers, and sequences (available via the Genbank accession numbers) used to create the custom probeset for LGC Rapid Genomics’ Capture-seq pipeline.** Unavailable sequence data is indicated by “—“.

| Species | ITS | rBCL | *trn*T–*trn*L | *trn*L–*trn*F | *psb*A–*trn*H | *ndh*F | *mat*K | *rp*10 | *ycf*1 |
| --- | --- | --- | --- | --- | --- | --- | --- | --- | --- |
| *Acrocomia aculeata* (Jacq.) Lodd. ex Mart. | HQ265478.1 | AY044625.1 | — | HQ265759.1 | KJ426591.1 | AY044555.1 | AM114639.1 | — | — |
| *Agave americana* L. | — | — | — | — | — | — | JX903544.1 | GQ402511.1 | — |
| *Agave celsii* Hook. | — | — | — | — | — | AF508398.1 | — | — | — |
| *Agave fourcroydes* Lem. | — | — | — | — | — | — | — | — | — |
| *Agave lechuguilla* Torr. | — | — | EU092448.1 | KX444110.1 | — | — | — | — | — |
| *Agave sisalana* Perrine ex Engelm. | — | GU135234.1 | — | — | GU135405.2 | — | GU135070.1 | — | — |
| *Agave striata* Zucc. | AM884825.1 | — | — | — | — | — | — | — | — |
| *Amaranthus albus* L. | — | — | — | KP318863.1 | — | — | — | — | — |
| *Amaranthus cruentus* L. | MG685226.1 | MF135386.1 | — | — | — | — | — | — | — |
| *Amaranthus hybridus* L. | — | — | — | — | MF143703.1 | — | MF159449.1 | — | — |
| *Amaranthus hypochondriacus* L. | — | X51964.1 | — | — | MF143751.1 | — | MG685144.1 | — | — |
| *Amaranthus quitensis* Kunth | — | — | — | — | — | AF194822.1 | — | — | — |
| *Amaranthus* sp. | — | — | LT996928.1 | — |  | — | — | — | — |
| *Anacardium occidentale* L. | AB071690.1 | AY462008.1 | — | AY594497.1 | KR075990.1 | KF664342.1 | AY594459.1 | — | — |
| *Ananas comosus* (L.) Merr. | — | L19977.1 | — | KU762973.1 | AB331266.1 | AY147766.1 | KU258039.1 | — | KU096009.1 |
| *Ananas nanus* (L.B.Sm.) L.B.Sm. | — | — | DQ084662.1 | — | — | — | — | — | — |
| *Annona glabra* L. | — | — | — | — | — | — | — | — | GU937365.1 |
| *Annona muricata* L. | — | AY743440.1 | DQ861648.1 | AY145352.1 | AY841428.1 | EF179282.1 | AF543722.1 | — | — |
| *Attalea allenii* H.E.Moore | — | AJ404829.1 | — | — | — | — | — | — | — |
| *Attalea cohune* Mart. | — | — | — | — | — | — | — | — | KU323514.1 |
| *Attalea phalerata* Mart. ex Spreng. | HQ265688.1 | — | — | HQ265783.1 | — | — | — | — | — |
| *Attalea rostrata* Oerst. | — | — | — | — | GQ982156.1 | — | — | — | — |
| *Bactris gasipaes* Kunnth | HQ265506.1 | AM110214.1 | — | DQ445909.1 | — | EU004890.1 | HQ265554.1 | — | — |
| *Bactris humilis* (Wallace) Burret | — | — | — | — | — | AY044558.1 | — | — | — |
| *Bactris major* Jacq. | — | GQ981678.1 | — | — | HG963669.1 | — | HQ265556.1 | — | — |
| *Bixa orellana* L. | — | AF022128.1 | — | FM179540.1 | HG963562.1 | EU077547.1 | FM179929.1 | — | — |
| *Brasenia schreberi* J.F.Gmel. | AY858636.1 | KX527460.1 | AY145329.1 | AM489713.1 | AB331296.1 | — | AF092973.1 | — | — |
| *Brosimum alicastrum* Sw. | — | AF500346.1 | — | AF501601.1 | HG963667.1 | AY289328.1 | GQ981947.1 | — | — |
| *Brosimum guianense* (Aubl.) Huber | AY635492.1 | — | — | — | — | — | — | — | — |
| *Byrsonima crassifolia* (L.) Kunth | — | AB233898.1 | — | AF350945.1 | GQ429122.1 | AF351011.1 | AF344535.1 | — | — |
| *Byrsonima* sp. | KJ123874.1 | — | — | — | — | — | — | — | — |
| *Cabomba aquatica* Aubl. | — | JX100671.1 | — | — | JX100499.1 | — | — | — | — |
| *Calathea allouia* (Aubl.) Lindl. | — | — | — | — | — | — | — | — | — |
| *Calathea crotalifera* S.Watson | — | AY656112.1 | — | — | — | AY656083.1 | — | — | — |
| *Calathea lutea* (Aubl.) Schult. | JQ341287.1 | — | — | JQ341231.1 | GQ429113.1 | — | JQ341348.1 | — | — |
| *Canavalia bonariensis* Lindl. | AY293839.1 | — | — | — | — | — | — | — | — |
| *Canavalia ensiformis* (L.) DC. | — | U74238.1 | — | EU717354.1 | GU396816.1 | — | KT751481.1 | — | — |
| *Canna indica* L. | FJ939544.1 | HM849844.1 | — | AM113702.1 | AB331269.1 | FJ861155.1 | AM114724.1 | — | — |
| *Capsicum annuum* L. | — | KJ773334.1 | DQ077635.1 | AY348966.1 | JQ087869.1 | DQ667527.1 | EF537302.1 | — | — |
| *Capsicum chinense* Jacq. | — | AB721663.1 | DQ077660.1 | EU603443.1 | EF537226.1 | EU603442.1 | EF537315.1 | — | — |
| *Capsicum frutescens* L. | — | JX996068.1 | DQ082852.1 | AY348989.1 | JQ087871.1 | DQ667538.1 | EF537312.1 | — | — |
| *Capsicum pubescens* Ruiz& Pav. | AY875749.1 | — | — | — | — | — | — | — | — |
| *Carica papaya* L. | AY461547.1 | JX091914.1 | — | JX091823.1 | KC867738.1 | AY483248.1 | AY483221.1 | — | — |
| *Chamaedorea tepejilote* Liebbm. | — | GQ981699.1 | — | AM497773.1 | GQ982181.1 | DQ273108.1 | DQ178691.1 | — | — |
| *Chenopodium acuminatum* Willd. | — | — | — | — | — | AY858613.1 | — | — | — |
| *Chenopodium berlandieri* Moq. | KP226664.1 | MG249740.1 | — | HE577567.1 | — | — | HE855645.1 | — | — |
| *Chenopodium ficifolium* Sm. | — | — | — | — | MF143759.1 | — | — | — | — |
| *Crescentia alata* Kunth | — | — | — | — | — | FJ887857.1 | — | — | — |
| *Crescentia cujete* L. | — | KJ082242.1 | AY500412.1 | — | KJ426683.1 | — | JQ587002.1 | — | — |
| *Cucurbita argyrosperma* K.Koch | — | HQ438617.1 | — | HQ438663.1 | — | — | HQ438592.1 | — | — |
| *Cucurbita ficifolia* Bouché | — | HQ438631.1 | — | HQ438677.1 | — | — | HQ438599.1 | — | — |
| *Cucurbita moschata* Duchesne | FJ915111.3 | HQ438614.1 | — | HQ438660.1 | — | — | HQ438603.1 | — | — |
| *Cucurbita pepo* L. | KT347507.1 | L21938.1 | — | HQ438672.1 | — | — | HQ438611.1 | — | — |
| *Datura innoxia* Mill. | AY875750.1 | — | — | — | — | — | — | — | — |
| *Datura stramonium* L. | — | — | — | EU580984.1 | — | EU580875.1 | KP756825.1 | — | — |
| *Dioscorea alata* L. | KJ956698.1 | — | — | — | — | — | — | — | — |
| *Dioscorea bulbifera* L. | — | — | — | — | — | AY007652.2 | — | — | — |
| *Dioscorea trifida* L.f. | — | KM877929.1 | D89681.1 | D89683.1 | KR072380.1 | — | KR072314.1 | — | — |
| *Elaeis oleifera* (Kunth) Cortés | HQ265521.1 | AY012509.1 | — | HQ265802.1 | GQ982210.1 | AY044562.1 | HQ265568.1 | — | — |
| *Gossypium barbadense* L. | GU935141.1 | AF031447.1 | AF031435.1 | — | HM437884.1 | U55339.1 | JN201361.1 | — | — |
| *Gossypium hirsutum* L. | KC404827.1 | M77700.1 | AF031434.1 | HQ696725.1 | HM437901.1 | U55340.1 | AY321158.1 | — | AY800381.1 |
| *Helianthus annuus* L. | KX671853.1 | L13929.1 | AY215931.1 | KM385516.1 | JF321291.1 | AB530934.1 | AY215805.1 | — | — |
| *Indigofera circinella* Baker f. | — | — | — | KR738686.1 | — | — | — | — | — |
| *Indigofera kirilowii* Maxim. ex Palib. | — | — | — | — | — | — | — | — | KP088125.1 |
| *Indigofera suffruticosa* Mill. | AF467051.1 | KX119304.1 | — |  | HG963791.1 |  | AF142697.1 | — | — |
| *Ipomoea batatas* (L.) Lam. | KC621864.1 | AY942199.1 | — | AY101071.1 |  | AF130177.1 | AJ429355.1 | — | — |
| *Ipomoea marabaensis* D.F.Austin & Secco | — | — | — | — | MF172049.1 | — | — | — | — |
| *Ipomoea sp.* | — | — | — | — | — | — | — | GQ402503.1 | — |
| *Lagenaria siceraria* (Molina) Standl. | KM051438.1 | DQ535825.1 | — | DQ536771.1 | GQ248323.1 | — | DQ536694.1 | — | — |
| *Lycopersicon esculentum* Mill. | — | L14403.1 | DQ180450.1 | DQ180450.1 | GU562406.1 | U08921.1 | — | — | — |
| *Manihot esculenta* Crantz | JQ743203.1 | LT576833.1 | — | EU518905.1 | KP692112.1 | — | GU214863.1 | — | — |
| *Manilkara zapota* (L.) P.Royen | KF686242.1 | EU980807.1 | — | DQ924309.1 | — | AY230696.1 | DQ924092.1 | — | KP088426.1 |
| *Maranta arundinacea* L. | JQ341260.1 | JQ592612.1 | — | AY140384.1 | HG963784.1 | — | JQ341325.1 | — | — |
| *Maranta leuconeura* É.Morren | — | — | — | — | — | — | — | GQ402513.1 | — |
| *Maranta bicolor* Ker Gawl. | — | — | — | — | — | AY656094.1 | — | — | — |
| *Nicotiana rustica* L. | AJ492415.1 | MG221507.1 | — | — | — | AJ585935.1 | AB039992.1 | — | — |
| *Nicotiana tabacum* L. | AJ012367.1 | KC825342.1 | — | FJ490822.1 | FJ493313.1 | L14953.1 | KJ652184.1 | NM_001324800.1 | — |
| *Nymphaea ampla* (Salisb.) DC. | — | — | AM422044.1 | AM422044.1 | — | — | — | — | — |
| *Nymphaea odorata* Aiton | AY858641.1 | — | — | — | — | — | — | — | — |
| *Opuntia ficus-indica* (L.) Mill. | EU559669.1 | FJ026615.1 | — | — | FJ026613.1 | JF787380.1 | FN997314.1 | — | — |
| *Opuntia guatemalensis* Britton & Rose | — | — | — | — | — | — | JQ587177.1 | — | — |
| *Opuntia megarhiza* Rse | — | — | — | — | — | — | — | — | JN387175.1 |
| *Opuntia phaeacantha* Engelm. | — | — | AF432935.1 | — | — | — | — | — | — |
| *Opuntia tomentosa* Salm-Dyck | — | — | — | JF712836.1 | — | JF787451.1 | — | — | — |
| *Attalea speciosa* Mart. | — | — | — | — | — | AY044570.1 | — | — | — |
| *Pachyrhizus erosus* (L.) Urb. | AY293846.1 | EU717260.1 | — | EU717324.1 | GU396706.1 | — | EU717401.1 | — | — |
| *Persea americana* Mill. | KX509877.1 | AY337727.1 | JQ742021.1 | GU250776.1 | JQ513882.1 | JQ437545.1 | AJ247179.2 | — | — |
| *Persea* sp. | — | — | — | — | — | — | — | GQ402505.1 | — |
| *Phaseolus acutifolius* A.Gray | KF943736.1 | — | — | — | — | — | DQ445952.1 | — | — |
| *Phaseolus coccineus* L. | KF943726.1 | LT576851.1 | GQ411774.1 | JQ041849.1 | JX495464.1 | — | DQ445965.1 | — | CA910235.1 |
| *Phaseolus lunatus* L. | KF943740.1 | LT576852.1 | GQ411716.1 | GU126367.1 | FJ951179.1 | — | DQ445985.1 | — | — |
| *Phaseolus vulgaris* L. | — | KX119315.1 | GQ411777.1 | EU717342.1 | FJ951239.1 | — | DQ450862.1 | — | JZ715503.1 |
| *Physalis philadelphica* Lam. | — | MG222711.1 | — | EU581045.1 | — | EU580929.1 | EF438953.1 | — | — |
| *Piper auritum* Kunth | AF275175.1 | EF590560.1 | — | EU519625.1 | EU581473.1 | EU519715.1 | DQ882205.1 | — | NC_034697.1 |
| *Pistia stratiotes* L. | — | KC466591.1 | — | AY054706.1 | GU135357.2 | — | AY034182.1 | — | — |
| *Pouteria campechiana* (Kunth) Baehni | — | — | — | DQ344318.1 | — | — | — | — | — |
| *Pouteria gongrijpii* Eyma | — | — | — | — | FJ039074.2 | — | — | — | — |
| *Pouteria grandis* Eyma | — | JQ625857.2 | — | — | — | — | — | — | — |
| *Pouteria hispida* Eyma | DQ246691.1 | — | — | — | — | — | — | — | — |
| *Pouteria lucuma* (Ruiz & Pav). Kuntze | — | — | — | — | — | AY230725.1 | — | — | — |
| *Pouteria sapota* (Jacq.) H.E. Moore & Stearn | — | — | — | — | — | — | — | GQ402492.1 | — |
| *Prunus armeniaca* L. | — | — | — | — | — | — | — | — | KP089823.1 |
| *Prunus serotina* (Ehrh.) | HM453949.1 | DQ006123.1 | AM950169.1 | JX414453.1 | HQ596803.1 | JQ776954.1 | HQ235266.1 | — | — |
| *Psidium brownianum* DC. | — | — | — | MF954190.1 | — | — | — | — | — |
| *Psidium guajava* L. | AY487283.1 | KX527097.1 | — | — | JQ279707.1 | — | AB354958.1 | — | — |
| *Psidium guineense* Sw. | — | — | — | — | — | AY498809.1 | — | — | — |
| *Sechium edule* (Jacq.) Sw. | JN560209.1 | AY862553.1 | — | DQ536861.1 | JN560308.1 | — | DQ536727.1 | — | — |
| *Setaria macrostachya* Kunth | — | — | — | — | — | EU741956.1 | — | — | — |
| *Setaria parviflora* Kunth | KY968927.1 | KJ773886.1 | — | — | MF143749.1 | — | — | — | — |
| *Setaria viridis* (L.) P.Beauv. | — | — | JQ041827.1 | KX372490.1 | — | — | — | — | — |
| *Solanum lycopersicum* L. | KC213749.1 | — | — | — | — | — | — | AB518477.1 | — |
| *Spondias purpurea* L. | — | KU559308.1 | — | KR081868.1 | KJ026821.1 | — | KP774612.1 | — | — |
| *Spondias mombin* L. | AF080064.1 | — | — | — | — | — | — | — | — |
| *Theobroma cacao* L. | JQ228377.1 | AF022125.1 | — | HM488410.1 | MF348567.1 | AF287916.1 | AY321195.1 | — | — |
| *Vanilla planifolia* Andrews | AF391786.1 | JN181479.1 | — | AY557223.1 | MF348723.1 | — | JN181462.1 | — | JN181530.1 |
| *Xanthosoma sagittifolium* (L.) Schott | — | L10246.2 | — | AY555175.1 | — | — | EU886500.1 | — | — |
| *Zea mays* L. | DQ683016.1 | MG226097.1 | EF541347.1 | GQ870012.1 | AF543684.1 | U21985.1 | X86563.2 | NM_001359379.1 | — |
