# Supplementary material for: Psychoactive and other ceremonial plants from a 2,000-year-old Maya ritual deposit at Yaxnohcah, Mexico
Source: PLoS One. 2024 Apr 26;19(4):e0301497. doi: 10.1371/journal.pone.0301497 (PMC11051596; doi:10.1371/journal.pone.0301497)
Supplement: S4 Table — The following sequences were subjected to a BLAST search using the National Center for Biotechnology Information (NCBI) database. Unlike the four plants discussed in the main body of the paper, the plants listed in this table could not be resolved to the genus or species level, likely because the sequences we recovered were too highly conserved among plant taxa. The actual sequences for each plant listed here are followed by the results of the BLAST searches (S5 Table). (DOCX) [file pone.0301497.s004.docx]

**S4 Table. Vascular plant nucleotide sequences from Operation 18E9 of the Helena complex.** The following sequences were subjected to a BLAST search using the National Center for Biotechnology Information (NCBI) database. Unlike the four plants discussed in the main body of the paper, the plants listed in this table could not be resolved to the genus or species level, likely because the sequences we recovered were too highly conserved among plant taxa. The actual sequences for each plant listed here are followed by the results of the BLAST searches (S5 Table).

Plant #1. Assigned taxon: Poaceae

Sequence:

**CIN_120903_P001_WC03_NODE_101127_length_208_cov_14.183007**

GCTCCTGCTTCTTCAGGCGGAACCCCGAGCTGAGGAGTTACTCGGAATGCTGCCAAGATA

TCAGTATCCTTGGTTTCGTACTCCGGGGTGTAGTAAGTCAATTTATAATCCTTAACACCA

GCTTTAAATCCAACACTTGCTTTAGTTTCTGTTTGTGGTGACATAAGTCCCTCCCTACAA

CTCATGAATTAAGAATTCTCACAACGAC

Plant #2. Assigned Taxon: Apocynaceae

Sequence:

**CIN_120903_P001_WC03_NODE_15752_length_410_cov_2.504225**

TGGTCGCTTTTCTTTCTTTTTCTTTTGAAAGCGGCATTCTCTCTTGACTCTAGAAACTCG

GACAATTATGCCATTCGGAAGAAGTCTTCTACAGAGGGAAAGCCTGTTACGAGTAAGTGG

AGAGAAAGATCTCCAGAGATTCTTATCTCATTCCATTCCAGTGGCTCAACCAGTAACCAA

TGGCGAAAACTCAAAAATCCATGGTTTCCCGGTAGAACCCTATTTCGCCCAAGTTGTTTC

GGAACCGGAAAAAAGAAGCGGTTTTTCGCACAGCTTGCTCATAGCGCAGGTCCCACTTGT

ATATCGTATTTGGCCGAAAAAGCATCAGACAGGTTGGAGTTCTTACCTTCTTGGGACTCC

ATGGACCAAGATCTGCTTTTATTATATGGTCAATACCGATCTACTTTAGT

Plant #3. Assigned Taxon: Moraceae

Sequence:

**CIN_120903_P001_WC03_NODE_2344_length_1857_cov_278.156493**

CGCTAAAAACGATATAAATAGGGAAATATATGGAATATGGAAAATATATATTTTAAGCAG

AATTATAATATTAGGTGTAATAATACTATACATTTATAGTATTATATATTTATATAATAC

TATACATTTATATATAATGTATATATAATATTATAATATCTGATATGTATCTATATATAT

TATATATTGATTTGTGGGTAGAGAAATAATAGAATCTTTTCTAATTGGATCAAATATGAG

TTTCCGAGAAAGATGAAAACAGATAGACAAGAAATCCAAATACCAAATATAAGTATAAGA

AAACAGTTTTCAATATGAGAACCACTATCTAATGAATTCAACAATTCCATCATAATATAA

ATGAAATAAAAAGGAAAAGGGTATCATAATGAAATCCTAATCACAAACCAAAAGGGGGAT

ATGGCGAAATTGGTAGACGCTACGGACTTAATTGAATTGAGCCTTGGTATGGAAACCTAC

CAAGTGATAACTTTCAAATTCAGAGAAACCCTGGAATTAAAAAAGGGCAATCCTGAGCCA

AATCCAGTTTTCTGAAAACAAACAAGGGTTCAGAAGGCGATAATAAAAAAGGATAGGTGC

AGAGACTCAATGGAAGCTGTTCTAACAAATGGAGTTGGCTGCGTTAGTAAAGGAATCACT

CCAGAAAGGATGAAGAATAAACCTATATACGTACTGAAATACTATCTTCAAACGATTAAT

GACAACCCAAATCCGTATTTCTTTTAATTTTCATGAAAAATTAAAGAATTGTTGTGAATC

AATTATAAGTTGAAAAAAGAATCAAATATTCATTGATCAAATCATTTACTACATCAAAAT

CTGATAGATCTTTTGAAGAATTGATTAATCGGACGAGAATAAAGATAGAGTCCCGTTCTA

CATGTCAATATCGACAACAATGAAATTTATAGTAAGAGGAAAATCCGTCGACTTTAAAAA

TCGTGAGGGTTCAAGTCCCTCTATCCCCAAAAAGGCCCATTTGATTCCCTAATTATTTAT

CCTACCTTCTCATTTTGTTAGCAGTTCAAAATTCGTTATCTTTCTCGTTCATTCTAATTC

TACAAACGTATTTGATCGAAAATTTTTTTCTTATCACAAGCCTTGTGATTTATATGAAAC

ACGTACAAATGAACATCTTTGAGAAGGGAATCCCATGTTAAATTTGAATAATTAATAATT

CTTTTTATTACTCGTACTGTACTGAAACTTACAAAGTCTTTTTTTTGAAGATCCAAGAAA

TTCCACCAAGGCCTGGATAAGAATTAGCAATACTCCTTTCGTCTTTTTAATTGACATAGA

CCCAAGTCCTCTATTTAAATTAAAAATTAAAATGAGGATGATGTGTAAGGGATGGTCGGG

ATAGCTCAGCTGGTAGAGCAGAGGACTGAAAATCCTCGTGTCACCAGTTCAAATCTGGTT

CCTGGCAAATTTGTATGAGTATCTATTCTACAAATTAATTTATATGGGTCGACATGCATA

TTCATTAAATAGTATGAATTCATTAATTCATTAACATAGTATAAATTCATTAAATAGATC

ATGATACATATTTATCCATCTAAATATCTGGGGATGTACTGTACTCCTTCTATATTTATA

GAATATATATATAGTTAAAGATGAGTAAAGAGTATATACTCTTTTTGATATGTATAACAA

AGTATGTAAAAGAAAATATTAATACTTCATAGATATTACAAAAGGTAAGTTGAAAAACCT

AAAAGTCTAGTCTAGGGGAGTTAAGGGGTGCGAATAGCCAAGATTGATCTCCGATACAGT

ACAAATAGAATCTGATCCCCTTTCATTTCTTTCTATTTTATTTTCATAGATTATTTC

Plant #4. Assigned Taxon: Moraceae

Sequence:

**CIN_120903_P001_WC03_NODE_2713_length_1674_cov_3320.096356**

GAGGAAGGAGAAGTCGTAACAAGGTTTCCGTAGGTGAACCTGCGGAAGGATCATTGTCGA

AACCTGCCCAGCAGAAAGACCCGCGAACACGTTAGAAACACTCGGGGGGCACAGGGTGCC

AATGAGCCTCGAACCCCCTGTGCCTAGGGCTTCGCCCATGGTCGACGGCCTAGGCTTAAA

AACAAACCCCGGCGCGGAATGCGTCAAGGAAAAATGATGGAACGACCCGTGCCGCCACAG

CCCCGGGAAACGGTGCATTGCTGTGGTGGCTGATTCGTGATTGTTTTAAGTCAAAACGAC

TCTCGGCAACGGATATCTCGGCTCTCGCATCGATGAAGAACGTAGCGAAATGCGATACTT

GGTGTGAATTGCAGAATCCCGTGAACCATCGAGTCTTTGAACGCAAGTTGCGCCCGAAGC

CGTTTGGCCGAGGGCACGTCTGCCTGGGCGTCACACACCGTTGCCCCCCTAAACACCCCA

TGACACTCCCTGTTGGGTGTGTCTGGATTGATCAGGGGCAGAGGATGGCCTCCCGTGTGC

ACTTTGTCTCGCGGTTGGTCCAAAATTGAGTCCTCGGCCGCATTGCAGTGGCAATAGGTG

GTTGTCGATCATTCGGTGCCCCGTCACGTGTGTTGGCCTCGAATCGGGACTCGTGTAGAC

CCCAATGCATCACTTACGGATGCTTCCGATGCGACCCCAGGTCAGGCGGGGCTACCCGCT

GAGTTTAAGCATATCAATAAGCGGAGGAAAAGAAACTTACAAGGATTCCCCTAGTAACGG

CGAGCGAACCGGGAACAGCCCAGGTTGAGAATCGGGCGCCCTCGGCGTTCGAATTGTAGT

CTGAAGAAGCGTCCTCAGCGACGGACCGGGCCCAAGTCCCCTGGAAGGGGGCGCCGGAGA

GGGTGAGAGCCCCGTCGTGCCCGGACCCTGTCGCACCACGAGGCGCTGTCTGCGAGTCGG

GTTGTTTGGGAATGCAGCCCCAATCGGGCGGTAAATTCCGTCCAAGGCTAAATACTGGCG

AGAGACCGATAGCAAACAAGTACCGCGAGGGAAAGATGAAAAGGACTTTGAAAAGAGAGT

CAAACAGTGCTTGAAATTGTCGGGAGGGAAGCGGATGGGGGCCGGCGATGCGTCCTGGTC

GGATGTGGAACGGCGAGAGCCGGTCCGCCGATCGACTCGGGGCGTGGACCGATGCGGATT

GTTGCGGCGGCCCAAGCCCGGGCTGTTGAGATGCTCGTGGAGACGTCGTCGTAGCGATTG

TGGAAGGCAGCGCGCGCCGTCTCGGCGTGCCTTGGCATCTGCGCGCTCCTGGCATCGGCC

TGCGGGCTCCCCATTCGGCCCGTCTTGAAACACGGACCAAGGAGTCTGACATGTGTGCGA

GTCAACGGGTGAGAAAACCCGTAAGGCGAAAGGAAGCTGACTGGCGGGATCCCCTAGCGG

GTTGCACCGCCGACCGACCTTGATCTTCTGAGAAGGGTTCGAGTGAGAGCATGCCTGTCG

GGACCCGAAAGATGGTGAACTATGCCTGAGCGGGGCGAAGCCAGAGGAAACTCTGGTGGA

GGCCCGCAGCGATACTGACGTGCAAATCGTTCGTCTGACTTGGGTATAGGGGCGAAAGAC

TAATCGAACCGTCTAGTAGCTGGTTCCCTCCGAAGTTTCCCTCAGGATAGCTGG

Plant #5. Assigned Taxon: Moraceae

Sequence:

**CIN_120903_P001_WC03_NODE_3324_length_1429_cov_673.018923**

TAGATAATTAATTATCTTATACTTATAGTTACTTATAGTAGGCTCTCCATCTTGTGTCAA

ATTCGCATCGAAAAAAGAAATAATGGTTCTCTTCTATGAATCTTAAGAACTTTTTTCGTA

AAATCTCGCCTACTAAGCAGTTTATATTACAACACAGAATGAAAAGGACAATATACAAGA

TGGGTATAAGAAGTTGTGATATTCGGCTCGATCCATGATCCGAAACTGCAGGATATACAT

AGAAATAATACAAATAAATAAAAAAGAATACACCAATACAGAATACATCAATCCTAAGGA

TCCATAGCATAGGATTAATTGTGAATCCAACACAACAATAGAAAGATTTAAGTTGCTTCG

TCTTCTATTTTGTATTTAAGATCTTGTATATTAAGTATGTATCTATCAAAAATCTATACA

ATAGTATCTTTGTATTCGGCTCAATCCTTTTAGTAAAAGATTGGGCCGAGTTTAATTTCA

ATTCAAGATTCAATTAAGAATAAGAAATAAGAAAAGGAACAGTAAAGTTAAGTAGTTGCT

GGATTACAAAGTATCCACTGCCTCGAATTCAAATTTGATTTCCTTCCATACTTCACAAGC

AGCAGCTAGTTCAGGACTCCATTTACTAGCCTCACGAATAATTTCCCTCAATAGCAAGAT

CGCGTCCCTCATTACGAGCTTTTACACATGCTTCTAGAGCTACTCGATTAGCTACGGCAC

CGGGTGCATTTCCCCAAGGATGTCCTAAAGTTCCTCCACCGAATTGTAGTACGGCATCAT

CTCCAAAGATCTCGGTCAAAGCAGGCATATGCCAAACGTGAATACCCCCTGAAGCCACGG

GCAGAACACCTGGTAGAGAAACCCAATCTTGAGTGAAACCACGGCTTCGATCTTTTTCAA

TAAAATCATCACGTAGTAAATCAACAAATCCTAAAGTGATCTCTCTTTCCCCTTCAAGTT

TACCTACTACAGTACCTGCGTGAATATGATCTCCACCAGACAGACGTAAGGCTTTAGCTA

GTACGCGAAAGTGCATACCATGATTCTTCTGTCTATCAATAACTGCATGCATTGCACGAT

GGATATGAAGAAGTAGACCATTATCTCGACAATAATGAGCCAGGCTAGTATTTGCAGTGA

ATCCTCCTGTTAAGTAATCATGCATTATGATAGGAACTCCCGCAAATACAGCCCTTTTGA

TCATTTCTTCACATGTACCTGCAGTAGCATTCAAGTAATGTCCTTTGATTTCACCTGTTT

CAGCTTGTGCTTTATAAAGTGCTTCGGCACAAAATAAGAAACGGTCTCTCCAACGCATAA

ATGGTTGGGAATTCACGTTCTCATCATCTTTGGTAAAATCAAGTCCACCGCGAAGACATT

CATAAACTGCTCTACCGTAATTCTTAGCGGATAACCCCAATTTAGGTTT

Plant #6. Assigned Taxon: Moraceae

Sequence:

**CIN_120903_P001_WC03_NODE_4335_length_1136_cov_358.736355**

GACATTCATAAACTGCTCTACCGTAATTCTTAGCGGATAACCCCAATTTAGGTTTAATAG

TACATCCTAATAGGGGGCGGCCATACTTGTTCAATTTATCTCTCTCAACTTGGATACCAT

GGGGTGGTCCTTGGAAAGTTTTAGAATAAGCAGTAGGGATTCGCAAATCTTCCAGACGTA

GCGCACGCAAGGCCTTAAACCCAAATACATTACCCACAATGGAAGTAAACATGTTAGTAA

CAGAACCTTCTTCAAAAAGGTCTAAGGGGTAAGCTACATAAGCAATATATTGATTTTCTT

CTCCAGCAACGGGCTCGATGTTGTAGCATCGACCTTTGTAGCGATCAAGACTGGTAAGCC

CGTCAGTCCATACAGTTGTCCATGTACCAGTAGAAGATTCAGCAGCTACCGCAGCCCCTG

CTTCTTCAGGGGGAACTCCTGGTTGAGGAGTTACTCGAAATGCTGCCAAGATATCAGTAT

CTTTGACTTCATATTCAGGAGTGTAATAAGTCAATTTATAATCTTTAACACCAGCTTTGA

ATCCAACACTTGCTTTAGTCTCTGTTTGTGGTGACATAAGTCCCTCCCTACAACTCATGA

ATTAAGAATTCTCGCAATAACAAGGTCTACTCGACATGAATTAGGAGTTAATCAAACCTT

TCACAGGAATCTTTCACAAAATTATCAACTAAGATTATCAACTGATCAGAACGGTTCGTT

ATTAGACCGTGGTATTTGATTCGCCAAATACATCATTATTGTATACTTTTTCATATGTAT

AGCGCAACCCAACCCTTGTTTTTCAAGTTTCTAATCTCTTACCCCCCTTTTTTGAATCGA

AATATTTAAGAAATTCGCTCTTGACAGTAATATATGTTGTATATGTAAATCCTAGATGGA

AAAATATGCGGAATTCGTCCATGAAAAGAAAGAAGGGGTATAGATATAAAAAAGAAAAAA

AAAGATAATAGCCTAGGTCTAAATCAATATGTTGAAATAGGAAATAAGAGCCGATGAAAT

AGAAAAAGGGTAAATGGAGTTCGGGTTCGAATTCCATAGATAATATGCATGGTATTAATG

ATAGGCAAATGAAAGGTTTTCTCAAGATTTTTATTCATCCACTTTTTTGAAAATGG

Plant #7. Assigned Taxon: Fabaceae

Sequence:

**CIN_120903_P001_WC03_NODE_56076_length_250_cov_7.174359**

CGGCTTTTAAGAGCGACTCCATTTTTTACACATTTTTATGAAGTAATTGGTTCGTCCATA

CCATCGATAGAGTTTGTAAGACCACGACTGATCCAGAAGGGAATGAATGGAAAAAGTAGC

ATGTCGTATCAATGGCGAATTTTAAAAATATTTCATTTTTATGGAATCCGGCCAAATTTT

TTGTTTGAATTTTTGGCTTGAAACAAAAAAAAATTCAGTTGGGTTTAATTAATAAAGGGA

TAGAGCTTGG

Plant #8. Assigned Taxon: Bignoniaceae

Sequence:

**CIN_120903_P001_WC03_NODE_66198_length_241_cov_1.016129**

GAGAAGATTGGTTACGTAGAAAGACCAAAATGGATTCATATTCACATACATGAGAATTAT

ATAAGAATAAGAATAATCTTTGATTTCTTTTTGAAAAAGAGAAACTGGCTTTCTTTGGAG

TAATAAGACTATTCCAATTACAATACTCGTTAAGAAAGAATCGTAATAAATGCAAAGAAG

AGGCATCTTTTACCCAGTATCGAAGAATTTGAACCAAGATTGCCACATGTATAGGGTGGG

G

Plant #9. Assigned Taxon: Moraceae

Sequence:

**CIN_120903_P001_WC03_NODE_750_length_3692_cov_431.863349**

GGAGATAAGTTATAGTTTTTTTATCATTCAGATCAAATACTGATTTTGATCTAATTATTT

ATCTTTGCTCTATCAATATGTACAATATATATAAATTAGTGCAATACGAAATAATAATGA

AAATGGAAATTTCCATTTTCATTTTTTGGATATTCATCCAATTTTACTTAAAAAGATTAA

TAAGAATTGCATTTTAAAATTTTACTAAAACTACTATTTTTTTTTTTCATCTTTTTCATT

CAACAGTAATTTGTTCATATCATTTGACCAATTTGAACCTCTTGATTTGAAATATTTAAA

ATAGTTGAAAAAGATTCAGAATAATTCTAAAATTATATATTTTATTTTGTCTATTATTAA

GTTTTTATTTTATTAACTGACCCCTTTCATTTCAAAAGAAATAAGAGCTGGTAAATCAGA

AACAATTAATTAAGATAAAAATAAAAAGAATTCTTTTTATTTATTTTTATGGAATATATA

TATCAATATTCATGGATTATAGCTTTCATCTCATTTCCAGTTCCTATATTAATAGGAGTA

GGACTTCTATTTTTTCCAACGGCAACAAAAGATCTTCGCCGTATGTGGGTTTTTCCAAGT

GTTTTATTGTTAAGTATAGTTATGCTTTTTTCAACCTATCTAACTATTCAACAAATAAAT

AACCCTTCTATCTATCTATCTATATGGTCTTGGACTCTAAATAATGACTTTTCTTTAGAA

TTTGGTTATTTGGTTGACCCACTTACTTGTATTATGTTAATATTAATTACTACTGTTGGA

ATTTTGGTTCTTATTTATAGTGACAATTATATGTCTCATGATCAGGGATATTTGAGATTT

TTTGCTTATATCATTTTTTTCACTACTTCAATGGTAGGATTAGTTACTAGTTCTAATCTG

ATACAAATTTATTTTTTTTGGGAATTGGTTGGAATGTGTTCTTATCTATTAATAGGTTTT

TGGTTCACACGACCTACTGCAGCAAATGCTTGTCAAAAAGCTTTTGTAACTAATCGCGTC

GGAGATTTTGGTTTATTATTAGGAATTTTAGGTTTTTATTGGATAACGGGTAGTTTAGGA

TTTCGGGATTTGTTTGAAATATTCAATAACTTGGTTTATAATAATGAAGTTAATTTTTTA

TTTGCTACTTTGTGTGCCTTTCTATTATTTGCTGGTGCAATTGCTAAATCTGCCCAATTT

CCCCTTCATGTATGGTTACCCGATGCTATGGAAGGGCCTACCCCTATTTCAGCCCTTATA

CATGCTGCTACTATGGTTGCGGCTGGAATTTTTCTTGTAGCTCGGCTTCTTCCGCTTTTT

ATAGTTATACCTTATATAATGAATCTAATAGCTTTGCTGGGGATAATAACATTACTTTTA

GGAGCCACTTTAGCCCTTGCTCAAAAGGATATTAAGAGGGGTTTAGCTTATTCAACAATG

TCTCAATTGGGTTATATGATGCTAGCTCTAGGTATGGGTTCTTATCGGGCTGCTTTGTTT

CATTTGATTACTCATGCTTATTCCAAAGCATTGTTGTTTTTAGGATCTGGATCTATTATT

CATTCAATGGAAACTTTTGTTGGATATTCTCCAGATAAAAGCCAGAATATGATTCTTATG

GGGGGGTTAAAAAAATATGTACCAATTACAAAAACATCTTTTTTATTAGGTACGCTTTCT

CTTTGTGGTATTCCACCTCTTGGATGTTTTTGGTCCAAAGATGAAATTCTTAATGATAGT

TGGTTGTATTCACGAATTTTTGCAATAATAGCTTTTTCCACAGCTGGATTAACTGCATTT

TATATGTTTCGGATCTATTTACTTACTTTTGAGGGACATTTAAATGTTTATTTTCAAACT

TACAGTGATAAAAAAAGAAGTTCTGTTTATTCAATATCTTTATGGGGTAGAGAAGAGCAA

AAGTGGATTAAAACAAAATCTCATTTATTACCTTTATTAACCATGAATAATAATAAAAGG

ACTTCTTTTTTTTTAAAAAAGAAATATCCAATTAATATAAATGTAAGAAGTATGAGGGGA

CCTATTATTAATATTCCTAATTTCGTTACTAAGAACATTTTCTCTTATCCTCATGAATCG

GACAATACTATGCTATTTCCTATGCTTGTATTAGGTCTATTTACTTTGTTCATTGGAATT

ATAGGAATTCCTTTCTTCAATCAATTCAATCAAGAAGGAATGCAGTTGGATATATTAACA

AAATTCTTAACTCCGTCTATAAACCTTTTATATCAAAATAAAAAGATTTTGATGAATTGG

GATTGGTATGAATTTCTAACAAATGCAATTTTTTCAGTCAGTATAGCTTCCTTCGGAATA

CTTATAGCGTCTTTTTTATATAATCCTGGTTATTCATCTTTACAAAATTTGAATTTATTT

AATTCATTTGTTAATGTTAAAGGTATTTCGAATAAACTGAAAATTTTTAGGGATAAAATA

CTAAATGTGACATATGATTGGTCATATAATCGAGGTTATATTGATTTTTTTTATGCAACA

TTTTTAATTCAAGGTATAAGAATATTGTCTAAACTAATTCATTTTTTTGATAGACAAGTA

ATTGATGGAATTACGAATGGGGTCGGTATTGCAAGTTTTTTCGTCGGAGAAGTTATTAAA

TATGTGGGTATTGGTCGAATCTCTTCCTATCTTTTAGTGTATGTATCTTATGTATTAATC

TTTTTATTAATTTTTACTCTTTTTTTTTCTTTAAATATTTCTAACTTCAAATTTTCTTGA

TTCCGATCCATATCCAGATAATAAGTTTCATAAACGGGTCTATTTTTATATCGTGTCTCT

TTAAAGTGGAATTCATCTTTTGTTTTTTCCTTTCCATTCACTCGTATCTCTTCCGTTTCA

TCGATTTTTTCCGGATCCTCCTTTTCTTCCGAAAAAAGGGAAGGAGAAGGATCTTCTTCG

GTGGATCCCTCTTGTTCCTGTTTAGTCCCCTTCGTTTCTGAAGTTTTTTCTACATCTGTT

TCTTTCTCACTTTCTTCCGTTTCTTTCAGTTTCTTAGTAAGAATGGGTGACGGTATTCTG

CCTAAATAGTAGACACAGGTAATAAATAATAGAATACTAAAGATTCGAGCCATAAAATTT

CTCAATTCTGACATAAGGTACTTATTAGATCTAATAGAATTGTTTTGCTGTATCCAGACT

ACTACCAATCCAACCCATTTCATGAATAAAATGTGACCAATTAACCAACCAACAAAACTA

CTTGTTACAAATAACATCTTGTTGTTGCATCGAAACATATAAATGTTGACTAATCTGACT

AACATTGAACTTGGTAAAATGAAATGGTTGAATAATTGAAAAATGAGATTATTCAGGAAT

ACACATTGAATGCTAAGATTACGCATTGAATTTCTGTTAGTATATCCATAATCAAAAAAG

TGTTTGTGATTGTTCCAGAAGAAATGAAACAAAAGATACGGTAGAGCTAGGACAGTTATT

GTATGAGGTCTACCCAATGCTAGATGCAGAGGCGCATAATAGATCGATATGAACATCATG

AGCTGTCCCGTAATAAAACCGGTTGTTGCTGATACTTTCTTCTCGGTTCCTTCTTCTCCT

TCTTCCATAACACGAGCTCGGAGAAGGAAGAGATAAGAGGGCCCTATGGAGAATGTGGTC

AGAAATCCATAATAGAGTCCGACCACAACGAC

Plant #10. Assigned Taxon: Sapindales

Sequence:

**CIN_120903_P001_WC03_NODE_77010_length_230_cov_17.125714**

GGATTTTCCTCTTACTATAAATTTCATTGTTGTCGGTATTGCTATTGACATGTAGAATGG

GACTCTATCTTTATTCTCGTCCGATTGATTAATAAGTTCTTCAAAAGAAAACATCGATCA

GACTATGGAGTGAATGATTTGATCAATGAATATTCGATTCTTTCTTCAACTTGGAATCGA

TTCACAACAATTCTTTTCTTTTTTTTCATAAAAAAAAGATACGGATTCTG

Plant #11. Assigned Taxon: Sapindaceae

Sequence:

**CIN_120903_P001_WC03_NODE_89551_length_219_cov_1.560976**

TTCGAAAGTTTTTTCTATTTCTACATCTGTTTCTTCCTCACTTTCCTCCCTTTCTTCCGT

TTCTGAGGTTTCTTTCACTTTCAGTTTCTTAGTAAAAATGGGTGACGGTATTCTGCCTAA

ATAGTAGATACAGGTAATAAATAAGAGAATACTAAAGATTCGAGCCATAGAATTTCTCAA

TTCTGACACAAGGGACTTATTAGATCGAATAAGTCCATT
