# Supplementary material for: Psychoactive and other ceremonial plants from a 2,000-year-old Maya ritual deposit at Yaxnohcah, Mexico
Source: PLoS One. 2024 Apr 26;19(4):e0301497. doi: 10.1371/journal.pone.0301497 (PMC11051596; doi:10.1371/journal.pone.0301497)
Supplement: S5 Table — A decision tree for interpreting BLAST results for our eDNA data was published previously [16]. In brief, our eDNA gene sequences were compared to the NCBI database and their BLAST program (using a BLASTN algorithm) evaluated the closest fit for our gene sequences by generating Max scores and E values (Expect value). Generally, the higher the Max score the closer the match of our unknown sequences to sequences from plant species stored in the database. The E value can be stated as the likelihood that a specific sequence alignment is a result of chance rather than a true biological relationship between the unknown sequence and the species represented in the reference sequence. Thus, a lower E value indicates a more significant alignment. In numerous cases, only one plant species would be listed with the highest Max score. If the plant identified was native to southeastern Mexico or was a known New World cultigen, then our identification was clear and definitive. Because gene sequences can be conserved among even distantly related plants, however, it was not uncommon to find more than one species with a top ranked Max score. When this occurred, we followed the published decision tree to decide if a species or genus identification could be determined. Note that in all cases, we took a conservative approach and assigned a broader taxon if there was any ambiguity. (DOCX) [file pone.0301497.s005.docx]

**S5 Table.** **BLAST results from eDNA data.** A decision tree for interpreting BLAST results for our eDNA data was published previously [16]. In brief, our eDNA gene sequences were compared to the NCBI database and their BLAST program (using a BLASTN algorithm) evaluated the closest fit for our gene sequences by generating Max scores and E values (Expect value). Generally, the higher the Max score the closer the match of our unknown sequences to sequences from plant species stored in the database. The E value can be stated as the likelihood that a specific sequence alignment is a result of chance rather than a true biological relationship between the unknown sequence and the species represented in the reference sequence. Thus, a lower E value indicates a more significant alignment. In numerous cases, only one plant species would be listed with the highest Max score. If the plant identified was native to southeastern Mexico or was a known New World cultigen, then our identification was clear and definitive. Because gene sequences can be conserved among even distantly related plants, however, it was not uncommon to find more than one species with a top ranked Max score. When this occurred, we followed the published decision tree to decide if a species or genus identification could be determined. Note that in all cases, we took a conservative approach and assigned a broader taxon if there was any ambiguity.

Plant #1. Assigned taxon: Poaceae, BLAST Results:

| **Scientific Name** | **Max Score** | **Total Score** | **Query Cover** | **E-value** | **% ID** | **Accession Length** | **Accession #** |
| --- | --- | --- | --- | --- | --- | --- | --- |
| Saccharum hybrid cultivar B4362 | 385 | 385 | 100% | 8.00E-103 | 100.00% | 141172 | BK010677.1 |
| Saccharum spontaneum | 385 | 385 | 100% | 8.00E-103 | 100.00% | 141185 | BK010676.1 |
| Miscanthus sacchariflorus | 385 | 385 | 100% | 8.00E-103 | 100.00% | 141361 | BK010674.1 |
| Saccharum perrieri | 385 | 385 | 100% | 8.00E-103 | 100.00% | 141071 | MN342163.1 |
| Iseilema membranaceum | 385 | 385 | 100% | 8.00E-103 | 100.00% | 139647 | MK593554.1 |
| Dichanthium aristatum | 385 | 385 | 100% | 8.00E-103 | 100.00% | 138088 | MK593549.1 |
| Cymbopogon citratus | 385 | 385 | 100% | 8.00E-103 | 100.00% | 139878 | MK593547.1 |
| Sorghum bicolor | 385 | 385 | 100% | 8.00E-103 | 100.00% | 140663 | MK348612.1 |
| Zea mays | 385 | 385 | 100% | 8.00E-103 | 100.00% | 140454 | MK348606.1 |
| Coix lacryma-jobi | 385 | 385 | 100% | 8.00E-103 | 100.00% | 140863 | MH558672.1 |
| Miscanthus x giganteus | 385 | 385 | 100% | 8.00E-103 | 100.00% | 141334 | MH820379.1 |
| Miscanthus sacchariflorus | 385 | 385 | 100% | 8.00E-103 | 100.00% | 141337 | MH820376.1 |
| Sorghum sudanense | 385 | 385 | 100% | 8.00E-103 | 100.00% | 140755 | NC_042790.1 |
| Sorghum propinquum | 385 | 385 | 100% | 8.00E-103 | 100.00% | 140642 | NC_042789.1 |
| Eriochrysis pallida | 385 | 385 | 100% | 8.00E-103 | 100.00% | 140382 | NC_042751.1 |
| Tripidium ravennae | 385 | 385 | 100% | 8.00E-103 | 100.00% | 141244 | NC_042735.1 |
| Tripidium ravennae | 385 | 385 | 100% | 8.00E-103 | 100.00% | 141244 | MH767451.1 |
| Eriochrysis pallida | 385 | 385 | 100% | 8.00E-103 | 100.00% | 140382 | MH767450.1 |
| Dichanthium annulatum | 385 | 385 | 100% | 8.00E-103 | 100.00% | 136805 | NC_042145.1 |
| Themeda triandra | 385 | 385 | 100% | 8.00E-103 | 100.00% | 138735 | MT505020.1 |
| Themeda triandra | 385 | 385 | 100% | 8.00E-103 | 100.00% | 138910 | MT505019.1 |
| Themeda triandra | 385 | 385 | 100% | 8.00E-103 | 100.00% | 138883 | MT505018.1 |
| Themeda triandra | 385 | 385 | 100% | 8.00E-103 | 100.00% | 138833 | MT505017.1 |
| Themeda triandra | 385 | 385 | 100% | 8.00E-103 | 100.00% | 138998 | MT505016.1 |
| Themeda triandra | 385 | 385 | 100% | 8.00E-103 | 100.00% | 138816 | MT505015.1 |
| Themeda triandra | 385 | 385 | 100% | 8.00E-103 | 100.00% | 138850 | MT505014.1 |
| Themeda triandra | 385 | 385 | 100% | 8.00E-103 | 100.00% | 138900 | MT505013.1 |
| Themeda tremula | 385 | 385 | 100% | 8.00E-103 | 100.00% | 138665 | MT505012.1 |
| Themeda strigosa | 385 | 385 | 100% | 8.00E-103 | 100.00% | 139085 | MT505011.1 |
| Themeda mooneyi | 385 | 385 | 100% | 8.00E-103 | 100.00% | 139865 | MT505010.1 |
| Themeda minor | 385 | 385 | 100% | 8.00E-103 | 100.00% | 139649 | MT505009.1 |
| Themeda huttonensis | 385 | 385 | 100% | 8.00E-103 | 100.00% | 139655 | MT505008.1 |
| Themeda cymbaria | 385 | 385 | 100% | 8.00E-103 | 100.00% | 139036 | MT505007.1 |
| Themeda arguens | 385 | 385 | 100% | 8.00E-103 | 100.00% | 138790 | MT505006.1 |
| Themeda anathera | 385 | 385 | 100% | 8.00E-103 | 100.00% | 138941 | MT505005.1 |
| Pseudanthistiria umbellata | 385 | 385 | 100% | 8.00E-103 | 100.00% | 137900 | MT505004.1 |
| Iseilema vaginiflorum | 385 | 385 | 100% | 8.00E-103 | 100.00% | 139635 | MT505003.1 |
| Iseilema prostratum | 385 | 385 | 100% | 8.00E-103 | 100.00% | 139806 | MT505002.1 |
| Iseilema membranaceum | 385 | 385 | 100% | 8.00E-103 | 100.00% | 139582 | MT505001.1 |
| Iseilema laxum | 385 | 385 | 100% | 8.00E-103 | 100.00% | 139580 | MT505000.1 |
| Iseilema hubbardii | 385 | 385 | 100% | 8.00E-103 | 100.00% | 139716 | MT504999.1 |
| Iseilema anthephoroides | 385 | 385 | 100% | 8.00E-103 | 100.00% | 139777 | MT504998.1 |
| Heteropogon ritchiei | 385 | 385 | 100% | 8.00E-103 | 100.00% | 139424 | MT504997.1 |
| Heteropogon fischerianus | 385 | 385 | 100% | 8.00E-103 | 100.00% | 135014 | MT504994.1 |
| Heteropogon contortus | 385 | 385 | 100% | 8.00E-103 | 100.00% | 135098 | MT504993.1 |
| Heteropogon contortus | 385 | 385 | 100% | 8.00E-103 | 100.00% | 135073 | MT504992.1 |
| Heteropogon contortus | 385 | 385 | 100% | 8.00E-103 | 100.00% | 134998 | MT504991.1 |
| Heteropogon contortus | 385 | 385 | 100% | 8.00E-103 | 100.00% | 134983 | MT504990.1 |
| Heteropogon contortus | 385 | 385 | 100% | 8.00E-103 | 100.00% | 134979 | MT504989.1 |
| Heteropogon contortus | 385 | 385 | 100% | 8.00E-103 | 100.00% | 134988 | MT504988.1 |
| Heteropogon contortus | 385 | 385 | 100% | 8.00E-103 | 100.00% | 135001 | MT504987.1 |
| Heteropogon contortus | 385 | 385 | 100% | 8.00E-103 | 100.00% | 134979 | MT504986.1 |
| Heteropogon contortus | 385 | 385 | 100% | 8.00E-103 | 100.00% | 134980 | MT504985.1 |
| Heteropogon contortus | 385 | 385 | 100% | 8.00E-103 | 100.00% | 134991 | MT504984.1 |
| Heteropogon contortus | 385 | 385 | 100% | 8.00E-103 | 100.00% | 134885 | MT504983.1 |
| Heteropogon contortus | 385 | 385 | 100% | 8.00E-103 | 100.00% | 134988 | MT504982.1 |
| Heteropogon contortus | 385 | 385 | 100% | 8.00E-103 | 100.00% | 135040 | MT504981.1 |
| Heteropogon contortus | 385 | 385 | 100% | 8.00E-103 | 100.00% | 135092 | MT504980.1 |
| Heteropogon contortus | 385 | 385 | 100% | 8.00E-103 | 100.00% | 135112 | MT504979.1 |
| Heteropogon contortus | 385 | 385 | 100% | 8.00E-103 | 100.00% | 134987 | MT504978.1 |
| Heteropogon contortus | 385 | 385 | 100% | 8.00E-103 | 100.00% | 134979 | MT504977.1 |
| Heteropogon contortus | 385 | 385 | 100% | 8.00E-103 | 100.00% | 135078 | MT504976.1 |
| Heteropogon contortus | 385 | 385 | 100% | 8.00E-103 | 100.00% | 134979 | MT504975.1 |
| Heteropogon contortus | 385 | 385 | 100% | 8.00E-103 | 100.00% | 134991 | MT504974.1 |
| Heteropogon contortus | 385 | 385 | 100% | 8.00E-103 | 100.00% | 134979 | MT504973.1 |
| Heteropogon contortus | 385 | 385 | 100% | 8.00E-103 | 100.00% | 135095 | MT504972.1 |
| Eremopogon tuberculatus | 385 | 385 | 100% | 8.00E-103 | 100.00% | 139648 | MT504971.1 |
| Eremopogon foveolatus | 385 | 385 | 100% | 8.00E-103 | 100.00% | 140219 | MT504970.1 |
| Schizachyrium delavayi | 385 | 385 | 100% | 8.00E-103 | 100.00% | 100.00% | MT504969.1 |
| Cymbopogon schoenanthus | 385 | 385 | 100% | 8.00E-103 | 100.00% | 140062 | MT504968.1 |
| Cymbopogon obtectus | 385 | 385 | 100% | 8.00E-103 | 100.00% | 139704 | MT504966.1 |
| Cymbopogon densiflorus | 385 | 385 | 100% | 8.00E-103 | 100.00% | 551622 | MT504965.1 |
| Tripsacum laxum | 385 | 385 | 100% | 8.00E-103 | 100.00% | 140556 | MW387499.1 |
| Miscanthus sacchariflorus | 385 | 385 | 100% | 8.00E-103 | 100.00% | 673349 | LS975134.1 |
| Miscanthus oligostachyus | 385 | 385 | 100% | 8.00E-103 | 100.00% | 140864 | LS398102.1 |
| Chrysopogon zizanioides | 385 | 385 | 100% | 8.00E-103 | 100.00% | 140864 | MN635785.1 |
| Coix lacryma-jobi var. puellarum | 385 | 385 | 100% | 8.00E-103 | 100.00% | 140863 | MT471102.1 |
| Coix lacryma-jobi var. ma-yuen | 385 | 385 | 100% | 8.00E-103 | 100.00% | 300848 | MT471101.1 |
| Coix lacryma-jobi var. puellarum | 385 | 385 | 100% | 8.00E-103 | 100.00% | 139107 | MT471098.1 |
| Coix lacryma-jobi var. ma-yuen | 385 | 385 | 100% | 8.00E-103 | 100.00% | 7217 | MT471096.1 |
| Coix lacryma-jobi var. maxima | 385 | 385 | 100% | 8.00E-103 | 100.00% | 13390 | MT471095.1 |
| Coix lacryma-jobi var. stenocarpa | 385 | 385 | 100% | 8.00E-103 | 100.00% | 21163 | MT471094.1 |
| Saccharum x Tripidium arundinaceum | 385 | 385 | 100% | 8.00E-103 | 100.00% | 25542 | MT821853.1 |
| Eremochloa ophiuroides | 385 | 385 | 100% | 8.00E-103 | 100.00% | 14271 | MT806102.1 |
| Zea mays | 385 | 385 | 100% | 8.00E-103 | 100.00% | 2221 | XM_035965460.1 |
| Zea mays | 385 | 385 | 100% | 8.00E-103 | 100.00% | 13668 | XM_035963854.1 |
| Zea mays | 385 | 385 | 100% | 8.00E-103 | 100.00% | 31386 | XM_035963790.1 |
| Zea mays | 385 | 385 | 100% | 8.00E-103 | 100.00% | 9030 | XM_035963783.1 |
| Zea mays | 385 | 385 | 100% | 8.00E-103 | 100.00% | 13390 | XM_035963780.1 |
| Zea mays | 385 | 385 | 100% | 8.00E-103 | 100.00% | 5058 | XM_035963774.1 |
| Zea mays | 385 | 385 | 100% | 8.00E-103 | 100.00% | 9917 | XM_035963766.1 |
| Zea mays | 385 | 385 | 100% | 8.00E-103 | 100.00% | 3085 | XR_004854397.1 |
| Zea mays | 385 | 385 | 100% | 8.00E-103 | 100.00% | 1384 | XM_035963761.1 |
| Zea mays | 385 | 385 | 100% | 8.00E-103 | 100.00% | 20145 | XM_035963743.1 |
| Zea mays | 385 | 385 | 100% | 8.00E-103 | 100.00% | 5058 | XM_035963725.1 |
| Zea mays | 385 | 385 | 100% | 8.00E-103 | 100.00% | 9917 | XM_035963715.1 |

Plant #2. Assigned Taxon: Apocynaceae, BLAST Results:

| **Scientific Name** | **Max**  **Score** | **Total Score** | **Query Cover** | **E-value** | **% ID** | **Accession Length** | **Accession #** |
| --- | --- | --- | --- | --- | --- | --- | --- |
| Rhazya stricta | 628 | 628 | 85% | 7.00E-176 | 98.87% | 548608 | KJ485850.1 |
| Asclepias syriaca | 623 | 623 | 85% | 3.00E-174 | 98.58% | 682498 | KF541337.1 |

Plant #3. Assigned Taxon: Moraceae, BLAST Results:

| **Scientific Name** | **Max Score** | **Total Score** | **Query Cover** | **E-value** | **% ID** | **Accession Length** | **Accession #** |
| --- | --- | --- | --- | --- | --- | --- | --- |
| Broussonetia kazinoki | 2353 | 2353 | 100% | 0 | 89.99% | 160841 | MH223642.1 |
| Broussonetia kazinoki x B. papyrifera | 2335 | 2335 | 100% | 0 | 89.80% | 160903 | MF496038.1 |

Plant #4. Assigned Taxon: Moraceae, BLAST Results:

| **Scientific Name** | **Max**  **Score** | **Total Score** | **Query Cover** | **E-value** | **% ID** | **Accession Length** | **Accession #** |
| --- | --- | --- | --- | --- | --- | --- | --- |
| Malaisia scandens | 2278 | 2278 | 100% | 0 | 91.23% | 5841 | MH135781.1 |
| Broussonetia papyrifera | 2146 | 2146 | 100% | 0 | 89.81% | 5850 | MH135784.1 |

Plant #5. Assigned Taxon: Moraceae, BLAST Results:

| **Scientific Name** | **MaxScore** | **Total Score** | **Query Cover** | **E-value** | **% ID** | **Accession Length** | **Accession #** |
| --- | --- | --- | --- | --- | --- | --- | --- |
| Broussonetia kazinoki | 2276 | 2276 | 100% | 0 | 95.40% | 160841 | MH223642.1 |
| Broussonetia monoica | 2270 | 2270 | 100% | 0 | 95.33% | 160777 | NC_047181.1 |

Plant #6. Assigned Taxon: Moraceae, BLAST Results:

| **Scientific Name** | **MaxScore** | **Total Score** | **Query Cover** | **E-value** | **% ID** | **Accession Length** | **Accession #** |
| --- | --- | --- | --- | --- | --- | --- | --- |
| Broussonetia papyrifera | 1832 | 1832 | 98% | 0 | 96.20% | 160121 | MH189570.1 |
| Malaisia scandens | 1831 | 1831 | 98% | 0 | 96.20% | 161313 | NC_047182.1 |
| Malaisia scandens | 1831 | 1831 | 98% | 0 | 96.20% | 161313 | MH189568.1 |

Plant #7. Assigned Taxon: Fabaceae, BLAST Results:

| **Scientific Name** | **MaxScore** | **Total Score** | **Query Cover** | **E-value** | **% ID** | **Accession Length** | **Accession #** |
| --- | --- | --- | --- | --- | --- | --- | --- |
| Fordia splendidissima | 440 | 440 | 100% | 2.00E-119 | 98.40% | 2656 | AF142718.1 |
| Pongamia pinnata | 427 | 427 | 100% | 2.00E-115 | 97.60% | 9231 | KY189093.1 |

Plant #8. Assigned Taxon: Bignoniaceae, BLAST Results:

| **Scientific Name** | **Max**  **Score** | **Total Score** | **Query Cover** | **E-value** | **% ID** | **Accession Length** | **Accession #** |
| --- | --- | --- | --- | --- | --- | --- | --- |
| Amphilophium cuneifolium | 418 | 418 | 100% | 9.00E-113 | 97.93% | 157070 | NC_042915.1 |
| Amphilophium chocoense | 418 | 418 | 100% | 9.00E-113 | 97.93% | 156951 | NC_042914.1 |
| Amphilophium cuneifolium | 418 | 418 | 100% | 9.00E-113 | 97.93% | 157070 | MK415794.1 |
| Amphilophium chocoense | 418 | 418 | 100% | 9.00E-113 | 97.93% | 156951 | MK415793.1 |
| Amphilophium parkeri | 418 | 418 | 100% | 9.00E-113 | 97.93% | 1536 | MG749185.1 |
| Amphilophium nunezii | 418 | 418 | 100% | 9.00E-113 | 97.93% | 1536 | MG749181.1 |
| Amphilophium magnoliifolium | 418 | 418 | 100% | 9.00E-113 | 97.93% | 1536 | MG749180.1 |
| Amphilophium elongatum | 418 | 418 | 100% | 9.00E-113 | 97.93% | 1536 | MG749173.1 |
| Amphilophium dasytrichum | 418 | 418 | 100% | 9.00E-113 | 97.93% | 1536 | MG749169.1 |
| Amphilophium cuneifolium | 418 | 418 | 100% | 9.00E-113 | 97.93% | 1536 | MG749168.1 |
| Amphilophium cremersii | 418 | 418 | 100% | 9.00E-113 | 97.93% | 1536 | MG749166.1 |
| Amphilophium chocoense | 418 | 418 | 100% | 9.00E-113 | 97.93% | 1536 | MG749165.1 |
| Amphilophium arenarium | 418 | 418 | 100% | 9.00E-113 | 97.93% | 1536 | MG749158.1 |
| Dolichandra cynanchoides | 418 | 418 | 100% | 9.00E-113 | 97.93% | 158110 | NC_037460.1 |
| Dolichandra cynanchoides | 418 | 418 | 100% | 9.00E-113 | 97.93% | 158110 | MG831874.1 |
| Bignonia capreolata | 418 | 418 | 100% | 9.00E-113 | 97.93% | 1739 | HQ384518.1 |
| Markhamia lutea | 412 | 412 | 100% | 4.00E-111 | 97.51% | 770 | MN370401.1 |

Plant #9, Assigned Taxon: Moraceae. BLAST Results:

| **Scientific Name** | **MaxScore** | **Total Score** | **Query Cover** | **E-value** | **% ID** | **Accession Length** |  | **Accession #** |
| --- | --- | --- | --- | --- | --- | --- | --- | --- |
| Malaisia scandens | 6013 | 7754 | 100% | 0 | 96.12% | 161313 |  | NC_047182.1 |
| Malaisia scandens | 6013 | 7754 | 100% | 0 | 96.12% | 161313 |  | MH189568.1 |
| Broussonetia kazinoki x  B. papyrifera | 5975 | 7726 | 99% | 0 | 96.00% | 160903 |  | MF496038.1 |

Plant #10. Assigned Taxon: Sapindales. BLAST Results:

| **Scientific Name** | **Max Score** | **Total Score** | **Query Cover** | **E-value** | **% ID** | **Accession Length** | **Accession #** |
| --- | --- | --- | --- | --- | --- | --- | --- |
| Tetragastris panamensis | 425 | 425 | 100% | 5.00E-115 | 100.00% | 846 | MK797675.1 |
| Tetragastris altissima | 425 | 425 | 100% | 5.00E-115 | 100.00% | 898 | MK797674.1 |
| Protium sagotianum | 425 | 425 | 100% | 5.00E-115 | 100.00% | 922 | MK797561.1 |
| Protium sagotianum | 425 | 425 | 100% | 5.00E-115 | 100.00% | 923 | MK797560.1 |
| Protium sagotianum | 425 | 425 | 100% | 5.00E-115 | 100.00% | 920 | MK797559.1 |
| Protium sagotianum | 425 | 425 | 100% | 5.00E-115 | 100.00% | 912 | MK797558.1 |
| Protium sagotianum | 425 | 425 | 100% | 5.00E-115 | 100.00% | 922 | MK797557.1 |
| Protium sagotianum | 425 | 425 | 100% | 5.00E-115 | 100.00% | 915 | MK797556.1 |
| Protium plagiocarpium | 425 | 425 | 100% | 5.00E-115 | 100.00% | 869 | MK797555.1 |
| Protium opacum | 425 | 425 | 100% | 5.00E-115 | 100.00% | 923 | MK797554.1 |
| Protium opacum | 425 | 425 | 100% | 5.00E-115 | 100.00% | 920 | MK797553.1 |
| Protium opacum | 425 | 425 | 100% | 5.00E-115 | 100.00% | 919 | MK797552.1 |
| Protium gallicum | 425 | 425 | 100% | 5.00E-115 | 100.00% | 924 | MK797550.1 |
| Protium gallicum | 425 | 425 | 100% | 5.00E-115 | 100.00% | 917 | MK797549.1 |
| Protium gallicum | 425 | 425 | 100% | 5.00E-115 | 100.00% | 923 | MK797548.1 |
| Protium decandrum | 425 | 425 | 100% | 5.00E-115 | 100.00% | 918 | MK797544.1 |
| Protium decandrum | 425 | 425 | 100% | 5.00E-115 | 100.00% | 926 | MK797543.1 |
| Protium decandrum | 425 | 425 | 100% | 5.00E-115 | 100.00% | 910 | MK797542.1 |
| Protium decandrum | 425 | 425 | 100% | 5.00E-115 | 100.00% | 917 | MK797541.1 |
| Protium altsonii | 425 | 425 | 100% | 5.00E-115 | 100.00% | 926 | MK797537.1 |
| Matayba sp. Stefano 245 | 425 | 425 | 100% | 5.00E-115 | 100.00% | 940 | KR081729.1 |
| Protium pilosum | 425 | 425 | 100% | 5.00E-115 | 100.00% | 865 | KJ503809.1 |
| Protium cranipyrenum | 425 | 425 | 100% | 5.00E-115 | 100.00% | 742 | KJ503808.1 |
| Tetragastris varians | 425 | 425 | 100% | 5.00E-115 | 100.00% | 832 | KJ503807.1 |
| Protium sp. Daly 13820 | 425 | 425 | 100% | 5.00E-115 | 100.00% | 774 | KJ503806.1 |
| Protium colombianum | 425 | 425 | 100% | 5.00E-115 | 100.00% | 658 | KJ503805.1 |
| Protium amplum | 425 | 425 | 100% | 5.00E-115 | 100.00% | 748 | KJ503803.1 |
| Protium glomerulosum | 425 | 425 | 100% | 5.00E-115 | 100.00% | 860 | KJ503802.1 |
| Protium cubense | 425 | 425 | 100% | 5.00E-115 | 100.00% | 821 | KJ503797.1 |
| Protium baracoense | 425 | 425 | 100% | 5.00E-115 | 100.00% | 822 | KJ503796.1 |
| Protium fragrans | 425 | 425 | 100% | 5.00E-115 | 100.00% | 865 | KJ503795.1 |
| Protium maestrense | 425 | 425 | 100% | 5.00E-115 | 100.00% | 775 | KJ503794.1 |
| Protium subacuminatum | 425 | 425 | 100% | 5.00E-115 | 100.00% | 825 | KJ503793.1 |
| Protium hebetatum | 425 | 425 | 100% | 5.00E-115 | 100.00% | 865 | KJ503792.1 |
| Tetragastris catuaba | 425 | 425 | 100% | 5.00E-115 | 100.00% | 865 | KJ503790.1 |
| Protium sp. AmaLin tree 24-IX-3a | 425 | 425 | 100% | 5.00E-115 | 100.00% | 724 | KJ503788.1 |
| Tetragastris balsamifera | 425 | 425 | 100% | 5.00E-115 | 100.00% | 865 | KJ503786.1 |
| Protium kleinii | 425 | 425 | 100% | 5.00E-115 | 100.00% | 808 | KJ503784.1 |
| Protium glaziovii | 425 | 425 | 100% | 5.00E-115 | 100.00% | 865 | KJ503783.1 |
| Protium brasiliense | 425 | 425 | 100% | 5.00E-115 | 100.00% | 864 | KJ503781.1 |
| Protium icicariba | 425 | 425 | 100% | 5.00E-115 | 100.00% | 859 | KJ503778.1 |
| Protium pittieri | 425 | 425 | 100% | 5.00E-115 | 100.00% | 865 | KJ503775.1 |
| Protium paniculatum var. riedelianum | 425 | 425 | 100% | 5.00E-115 | 100.00% | 865 | KJ503768.1 |
| Protium leptostachyum | 425 | 425 | 100% | 5.00E-115 | 100.00% | 817 | KJ503766.1 |
| Protium puncticulatum | 425 | 425 | 100% | 5.00E-115 | 100.00% | 865 | KJ503765.1 |
| Tetragastris altissima | 425 | 425 | 100% | 5.00E-115 | 100.00% | 788 | KJ503763.1 |
| Protium sp. AmaLin | 425 | 425 | 100% | 5.00E-115 | 100.00% | 742 | KJ503761.1 |
| Protium calanense | 425 | 425 | 100% | 5.00E-115 | 100.00% | 814 | KJ503756.1 |
| Protium unifoliolatum | 425 | 425 | 100% | 5.00E-115 | 100.00% | 865 | KJ503755.1 |
| Protium giganteum | 425 | 425 | 100% | 5.00E-115 | 100.00% | 865 | KJ503754.1 |
| Protium trifoliolatum | 425 | 425 | 100% | 5.00E-115 | 100.00% | 866 | KJ503748.1 |
| Protium plagiocarpium | 425 | 425 | 100% | 5.00E-115 | 100.00% | 865 | KJ503747.1 |
| Protium copal | 425 | 425 | 100% | 5.00E-115 | 100.00% | 820 | KJ503745.1 |
| Protium paniculatum var. paniculatum | 425 | 425 | 100% | 5.00E-115 | 100.00% | 715 | KJ503741.1 |
| Protium aracouchini | 425 | 425 | 100% | 5.00E-115 | 100.00% | 821 | KJ503736.1 |
| Protium sp. Fine 1306 | 425 | 425 | 100% | 5.00E-115 | 100.00% | 856 | KJ503735.1 |
| Protium crassipetalum | 425 | 425 | 100% | 5.00E-115 | 100.00% | 865 | KJ503730.1 |
| Protium spruceanum | 425 | 425 | 100% | 5.00E-115 | 100.00% | 865 | KJ503729.1 |
| Protium ravenii | 425 | 425 | 100% | 5.00E-115 | 100.00% | 865 | KJ503726.1 |
| Protium sagotianum | 425 | 425 | 100% | 5.00E-115 | 100.00% | 865 | KJ503725.1 |
| Protium heptaphyllum | 425 | 425 | 100% | 5.00E-115 | 100.00% | 865 | KJ503724.1 |
| P. heptaphyllum subsp. Ulei | 425 | 425 | 100% | 5.00E-115 | 100.00% | 865 | KJ503723.1 |
| Protium glabrescens | 425 | 425 | 100% | 5.00E-115 | 100.00% | 865 | KJ503722.1 |
| Protium elegans | 425 | 425 | 100% | 5.00E-115 | 100.00% | 865 | KJ503721.1 |
| Protium altsonii | 425 | 425 | 100% | 5.00E-115 | 100.00% | 869 | KJ503720.1 |
| Protium panamense | 425 | 425 | 100% | 5.00E-115 | 100.00% | 780 | KJ503718.1 |
| Protium sessiliflorum | 425 | 425 | 100% | 5.00E-115 | 100.00% | 865 | KJ503717.1 |
| Protium confusum | 425 | 425 | 100% | 5.00E-115 | 100.00% | 821 | KJ503716.1 |
| Protium costaricense | 425 | 425 | 100% | 5.00E-115 | 100.00% | 734 | KJ503715.1 |
| Protium glaucescens | 425 | 425 | 100% | 5.00E-115 | 100.00% | 926 | HQ655783.1 |
| Protium carnosum | 425 | 425 | 100% | 5.00E-115 | 100.00% | 926 | HQ655782.1 |
| Tetragastris panamensis | 425 | 425 | 100% | 5.00E-115 | 100.00% | 926 | HQ655781.1 |
| Protium sagotianum | 425 | 425 | 100% | 5.00E-115 | 100.00% | 926 | HQ655780.1 |
| Protium ferrugineum | 425 | 425 | 100% | 5.00E-115 | 100.00% | 926 | HQ655764.1 |
| Tetragastris altissima | 425 | 425 | 100% | 5.00E-115 | 100.00% | 957 | GU246110.1 |
| Protium guianense | 425 | 425 | 100% | 5.00E-115 | 100.00% | 957 | GU246107.1 |
| Protium copal | 425 | 425 | 100% | 5.00E-115 | 100.00% | 957 | GU246106.1 |
| Tetragastris panamensis | 425 | 425 | 100% | 5.00E-115 | 100.00% | 1003 | AY594538.1 |
| Protium divaricatum | 425 | 425 | 100% | 5.00E-115 | 100.00% | 1003 | AY594532.1 |
| Protium pallidum | 425 | 425 | 100% | 5.00E-115 | 100.00% | 1003 | AY594531.1 |
| Protium decandrum | 425 | 425 | 100% | 5.00E-115 | 100.00% | 570 | FJ039336.1 |
| Protium opacum | 425 | 425 | 100% | 5.00E-115 | 100.00% | 596 | FJ039330.1 |
| Protium morii | 425 | 425 | 100% | 5.00E-115 | 100.00% | 571 | FJ039328.1 |
| Trattinnickia demerarae | 425 | 425 | 100% | 5.00E-115 | 100.00% | 572 | FJ039321.1 |
| Protium trifoliolatum | 425 | 425 | 100% | 5.00E-115 | 100.00% | 550 | FJ039269.1 |
| Protium trifoliolatum | 425 | 425 | 100% | 5.00E-115 | 100.00% | 602 | FJ039265.1 |
| Protium gallicum | 425 | 425 | 100% | 5.00E-115 | 100.00% | 565 | FJ039247.1 |
| Protium gallicum | 425 | 425 | 100% | 5.00E-115 | 100.00% | 573 | FJ039240.1 |
| Protium gallicum | 425 | 425 | 100% | 5.00E-115 | 100.00% | 604 | FJ039238.1 |
| Protium gallicum | 425 | 425 | 100% | 5.00E-115 | 100.00% | 574 | FJ039235.1 |
| Protium decandrum | 425 | 425 | 100% | 5.00E-115 | 100.00% | 585 | FJ039230.1 |
| Tetragastris altissima | 425 | 425 | 100% | 5.00E-115 | 100.00% | 575 | FJ039111.1 |
| Tetragastris altissima | 425 | 425 | 100% | 5.00E-115 | 100.00% | 582 | FJ039110.1 |
| Protium opacum | 425 | 425 | 100% | 5.00E-115 | 100.00% | 591 | FJ039108.1 |
| Protium morii | 420 | 420 | 100% | 2.00E-113 | 99.57% | 907 | MK797551.1 |

Plant #11. Assigned Taxon: Sapindaceae, BLAST Results:

| **Scientific Name** | **Max Score** | **Total Score** | **Query Cover** | **E-value** | **% ID** | **Accession Length** | **Accession #** |
| --- | --- | --- | --- | --- | --- | --- | --- |
| Dimocarpus longan | 405 | 811 | 100% | 7.00E-109 | 100.00% | 160793 | MK726005.1 |
| Dimocarpus longan | 405 | 811 | 100% | 7.00E-109 | 100.00% | 160833 | NC_037447.1 |
| Dimocarpus longan | 405 | 811 | 100% | 7.00E-109 | 100.00% | 160833 | MG214255.1 |
| Litchi chinensis | 405 | 811 | 100% | 7.00E-109 | 100.00% | 162524 | KY635881.1 |
| Litchi chinensis | 405 | 811 | 100% | 7.00E-109 | 100.00% | 162525 | MW067100.1 |
| Dimocarpus longan | 405 | 811 | 100% | 7.00E-109 | 100.00% | 160789 | MW067098.1 |
| Nephelium lappaceum | 405 | 811 | 100% | 7.00E-109 | 100.00% | 161321 | MT936934.1 |
| Nephelium lappaceum | 405 | 811 | 100% | 7.00E-109 | 100.00% | 161356 | NC_053699.1 |
| Sapindus mukorossi | 399 | 799 | 100% | 3.00E-107 | 99.54% | 160481 | KM454982.1 |
